# Tagging large CNV blocks in wheat boosts digitalization of germplasm resources by ultra-low-coverage sequencing

**DOI:** 10.1186/s13059-024-03315-6

**Published:** 2024-07-01

**Authors:** Jianxia Niu, Wenxi Wang, Zihao Wang, Zhe Chen, Xiaoyu Zhang, Zhen Qin, Lingfeng Miao, Zhengzhao Yang, Chaojie Xie, Mingming Xin, Huiru Peng, Yingyin Yao, Jie Liu, Zhongfu Ni, Qixin Sun, Weilong Guo

**Affiliations:** 1 Frontiers Science Center for Molecular Design Breeding, Key Laboratory of Crop Heterosis and Utilization, Beijing Key Laboratory of Crop Genetic Improvement, China Agricultural University, Beijing, 100193 China; 2 Sanya Institute of China Agricultural University, Sanya, 572025 China

**Keywords:** Wheat, Copy number variation, Digitalized fingerprinting, Introgression, Low-coverage sequencing

## Abstract

**Background:**

The massive structural variations and frequent introgression highly contribute to the genetic diversity of wheat, while the huge and complex genome of polyploid wheat hinders efficient genotyping of abundant varieties towards accurate identification, management, and exploitation of germplasm resources.

**Results:**

We develop a novel workflow that identifies 1240 high-quality large copy number variation blocks (CNVb) in wheat at the pan-genome level, demonstrating that CNVb can serve as an ideal DNA fingerprinting marker for discriminating massive varieties, with the accuracy validated by PCR assay. We then construct a digitalized genotyping CNVb map across 1599 global wheat accessions. Key CNVb markers are linked with trait-associated introgressions, such as the 1RS·1BL translocation and 2N^v^S translocation, and the beneficial alleles, such as the end-use quality allele *Glu-D1d* (Dx5 + Dy10) and the semi-dwarf *r-e-z* allele. Furthermore, we demonstrate that these tagged CNVb markers promote a stable and cost-effective strategy for evaluating wheat germplasm resources with ultra-low-coverage sequencing data, competing with SNP array for applications such as evaluating new varieties, efficient management of collections in gene banks, and describing wheat germplasm resources in a digitalized manner. We also develop a user-friendly interactive platform, WheatCNVb (http://wheat.cau.edu.cn/WheatCNVb/), for exploring the CNVb profiles over ever-increasing wheat accessions, and also propose a QR-code-like representation of individual digital CNVb fingerprint. This platform also allows uploading new CNVb profiles for comparison with stored varieties.

**Conclusions:**

The CNVb-based approach provides a low-cost and high-throughput genotyping strategy for enabling digitalized wheat germplasm management and modern breeding with precise and practical decision-making.

**Supplementary Information:**

The online version contains supplementary material available at 10.1186/s13059-024-03315-6.

## Background

Wheat is one of the most widely grown and consumed crops and provides 20% of the total protein and calories in human nutrition [1]. Accurate identification and evaluation of genomic polymorphism within wheat germplasm resources are crucial to enhancing breeding capacity and developing improved varieties with higher yields and resistance to biotic and abiotic stresses [2, 3]. Beyond single nucleotide polymorphisms (SNPs) and small InDels, there are extensive structural variations (SVs) at a large scale in the wheat genome, which includes gene presence/absence variations (PAVs), copy number variations (CNVs), and chromosomal translocations, serving as an important source of genetic diversity in the wheat breeding population [4–6]. Current methods for identifying SVs generally require high-quality genome assemblies, high sequencing depth, or long-read sequencing [7], while the high cost of sequencing hinders profiling SVs at a population level.

DNA-based markers have been widely used for describing varieties and assisting breeding [8]. Multiple types of molecular markers derived from genomic variations have been developed to assist genome-based breeding in wheat, such as simple sequence repeats (SSRs), amplified fragment length polymorphisms (AFLPs), and SNPs [9]. Hybridization-based and PCR-based markers were the earliest molecular markers, which are time-consuming and laborious in genotyping, thus were difficult to apply in large-scale population analysis [10]. High-throughput methods such as genotyping-by-sequencing (GBS)-based and array-based SNP genotyping techniques were utilized to identify SNP/InDel markers [11, 12], while these effective markers are still limited and the overall cost to genotype one sample is still high to be utilized in assisting the variety management and breeding design [13]. More stable and effective DNA markers and corresponding cost-efficient scanning methods are urgently needed for describing and exploiting wheat germplasm resources.

Common wheat is a typical allohexaploid crop, its genome is of considerable tolerance to large segmental deletions and duplications [14–16], and highly plastic to take in both intraspecific and interspecific introgressions [17]. Furthermore, the genomes of modern wheat germplasms have been shaped by introgression from wild relatives during domestication [18] and by distant hybridization during the modern breeding process [19]. Thus, the characterization of SVs in wheat is important for accurate genotyping of massive varieties. Reported cytogenetic and molecular methods for detecting SVs, such as fluorescence in situ hybridization (FISH) and genomic in situ hybridization (GISH), are limited in aspects of throughput and resolution and are primarily used to confirm known SVs [20, 21]. Thus, there is an urgent need for a high-throughput and cost-effective method to characterize and exploit SVs across diverse wheat varieties.

The advent of the pan-genomic era brings opportunities for detecting SVs among wheat varieties globally [15]. Current main strategies include directly comparing genome assemblies and inferring SVs from the mapping of high-coverage resequencing data against the reference genome [22, 23]. However, the high costs of sequencing-based strategies hinder the application in genotyping the SVs at a large-scale population level [7]. Recently, Keilwagen et al. demonstrated that the depth-of-coverage of GBS or low-coverage whole genome sequencing could be used for detecting large chromosomal variations [24]. However, there is still a lack of an accurate and cost-efficient method for characterizing depth-of-coverage variations with stable performance across wheat varieties, tackling the highly noisy signals introduced by low-coverage sequencing data.

Here, we identified a set of high-quality CNV block (CNVb) markers by tagging large CNV blocks from a worldwide collection of wheat resequencing data using a pan-genome reference, supporting accurate profiling of the CNVb markers across wheat varieties even at an ultra-low sequencing coverage. The link between in silico CNVb markers with key introgressions and benefit alleles associated with agronomic traits further adds value to the digitalization of wheat germplasm. A free-to-access and user-friendly web platform (http://wheat.cau.edu.cn/WheatCNVb/) was also developed to help access and utilize the CNVb markers. In summary, the in silico CNVb markers can serve as new-generation molecular markers facilitating the characterization of the germplasm resources and assisting the genomic breeding in crops with high accuracy and low cost.

## Results

### Pervasive large CNV blocks identified in wheat

**Fig. 1:**
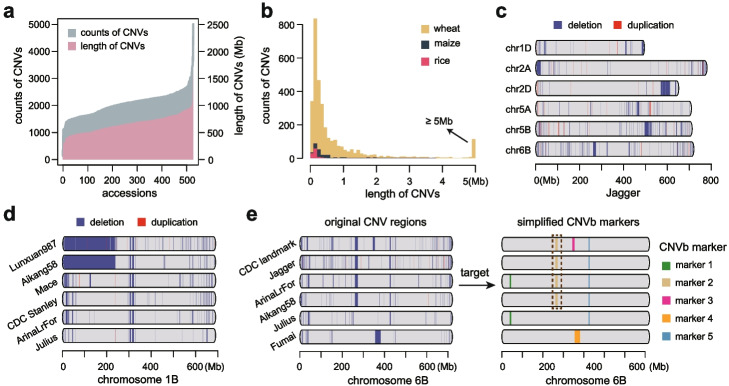
Characterization of large CNV blocks in wheat. **a** Distribution of total length and total count of CNV regions in each wheat accession. **b** Comparison of length and count of CNV regions between wheat, maize, and rice across the whole genome. **c** CNV region distribution of Jagger along chromosomes (1D, 2A, 2D, 5A, 5B, and 6B). Bin size, 100 Kb. **d** CNV region distribution on chromosome 1B among represent accessions (Lunxuan987, Aikang58, Mace, CDC Stanley, ArinaLrFor, and Julius). Bin size, 100 Kb. **e** Schematic representation of the conversion from the large CNV blocks (CNVb) (left) to tagged CNVb markers (right) on chromosome 6B. Left panel, CNV blocks with length ≥100 Kb. Right panel, CNVb markers, each color represents one unique CNVb marker. A particular CNV block (chr6B:265–278 Mb) was marked by a dashed rectangle. Bin size, 100 Kb

To comprehensively survey and characterize the genome-wide copy number variations (CNVs) in wheat, we collected a panel of whole genome resequencing data of worldwide wheat accessions [16, 17, 25–27], including 186 modern cultivars and 342 landraces (Additional file 1: Table S1). After mapping reads against the Chinese Spring (CS) reference genome IWGSCv1 [28], relative read depths were calculated bin-wisely with a bin size of 100 Kb. A total of 8430 Mb and 3375 Mb non-redundant bins were identified as deletion and duplication in at least one accession, respectively. The CNV bins were unevenly distributed across the genome, with higher frequencies observed near the ends of chromosomes (Additional file 2: Fig. S1), consistent with observations in maize [29] and rice [30]. Our results showed that the total length of CNV regions ranged from 139 to 1567 Mb across different varieties (Fig. 1a). Notably, the total lengths of CNV regions for 81.6% of the accessions exceed 500 Mb, with an average of 2061 CNV regions per accession, confirming that large CNV regions are pervasive across diverse wheat varieties. In contrast, maize and rice show fewer CNV regions, with average total lengths of 382 Kb and 142 Kb, and average counts of 53 and 19 CNV regions, respectively (Fig. 1b), highlighting the high-frequent and large CNV blocks as a unique feature of wheat.

As a key feature of CNV regions detected in wheat compared with rice or maize, there are more CNV regions exceeding one megabase. There are six extra-large CNV blocks with lengths spanning ≥10 Mb detected on chromosomes 1D, 2A, 2D, 5A, 5B, and 6B in the wheat cultivar Jagger (Fig. 1c). Many CNV blocks are shared among accessions, while positions and lengths of CNV blocks differ (Fig. 1d, e). Taking chromosome 1B as an example, a large CNVb-deletion (chr1B: 0–236.7 Mb) across 1BS can be observed in both Lunxuan987 and Aikang58 (Fig. 1d), which corresponds to the documented 1RS·1BL translocation [20]. Rather than the 1RS·1BL-related large CNV block presented in Lunxuan987 and Aikang58, several smaller CNV blocks were identified in Mace, CDC Stanley, ArinaLrFor, and Julius along the similar chromosome region (Fig. 1d), reflecting the diversity of CNV blocks on the genome. The consistent local CNV block landscapes observed among accessions may serve as ideal markers to identify genetic relationships of wheat resources. Thus, we proposed a strategy to tag intraspecific shared CNV blocks as in silico markers for wheat germplasm identification. As a prototype example, we selected the large CNV regions spanning chromosome 6B shared among CDC Landmark, Jagger, ArinaLrFor, and Aikang58, and grouped them into five CNVb markers, which could effectively compress the comprehensive CNV landscapes into a list of digitalized signals (Fig. 1e). The presence or absence of CNVb makers demonstrates an alternative and effective strategy in constructing the molecular fingerprints of wheat germplasm.

### Developing CNVb markers at pan-genome level

**Fig. 2:**
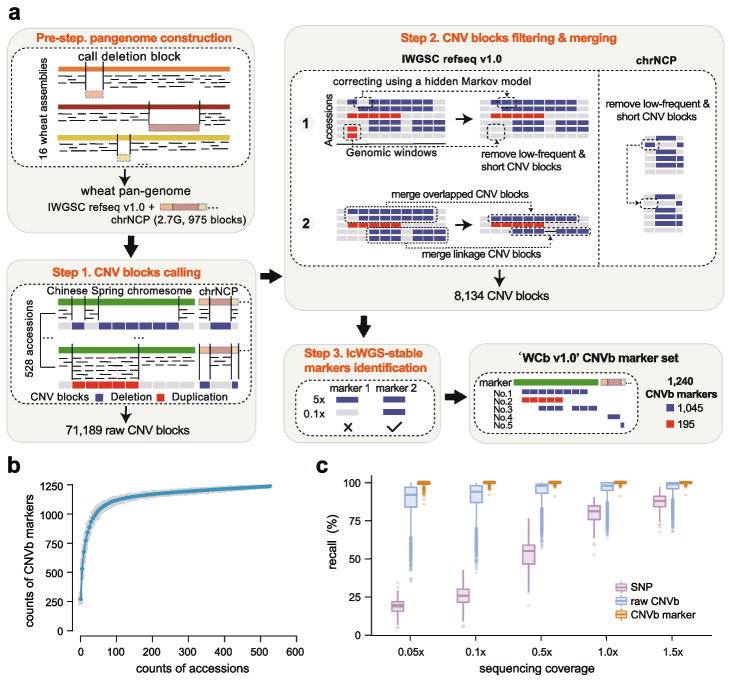
Development and evaluation of CNVb markers. **a** Pipeline to identify CNVb markers against wheat pan-genome. Pre-step, the pan-genome was constructed by combining the Chinese Spring assembly and the unmapped blocks of Chinese Spring relative to the other 16 wheat assemblies. Step 1, the initial CNV blocks of 528 high-quality wheat resequencing accessions were identified with a 100 Kb window. Step 2, a hidden Markov model (HMM) was introduced to smooth noisy signals, and then low frequency and short CNV blocks were filtered from the retained CNV blocks. CNV blocks with reciprocally overlapped regions larger than 80% were merged as a single CNVb cluster, and linkage clusters with close distances were further combined. Step 3, the final CNVb markers were extracted by eliminating those with low recall rates identified by ultra-low-coverage whole genome sequencing (ulcWGS), using CNVb markers identified by high-coverage whole genome sequencing as the ground truth. **b** Saturation analysis of CNVb markers. Five accessions were randomly added each time. The shaded area represents 100 replications for each sampling. The blue dot represents the average number of CNVb markers across 100 repetitions. **c** Comparison of the accuracy of SNPs, raw CNVs, and CNVb markers identified at low sequencing depth

To mitigate the potential bias of CNVb identification introduced by using a single reference genome and acquire sufficient markers, we constructed a pan-genome reference by iterative mapping of the whole-genome resequencing data against 16 de novo assembled reference genome sequences [15, 26, 31–35] (Additional file 1: Table S2). This iterative process involved using a 1 Mb sliding window to identify sequences present in genomes other than Chinese Spring (CS). Starting with the Aikang58 genome as the initial reference, and progressing through each genome in order of assembly quality, we systematically detected and compiled 975 novel genome blocks with a total length of 2.7 Gb (Additional file 1: Table S3). These blocks, which represent genomic regions absent in CS, were then assembled into a non-Chinese Spring pan-genome chromosome, denoted as “chrNCP” (Fig. 2a). The saturation analysis showed that the number of non-CS sequence blocks increased by adding new assemblies and approached a plateau when 14 genomes were included (Additional file 2: Fig. S2), indicating the representativeness of the constructed pan-genome by integrating a total of 17 wheat assemblies.

To identify representative and stable CNVb blocks for distinguishing wheat accessions, we developed a pipeline to obtain high-quality CNVb markers by examining consistent borders, overlaping ratio, and continuity of CNV blocks based on a panel of whole genome resequencing data with an average coverage of 5.4×, which covers 528 wheat accessions (Additional file 1: Table S1). The pipeline consists of three main steps: detecting raw CNV blocks, deducing low-confident and redundant CNV blocks, and removing CNV blocks sensitive to low sequencing depth (Fig. 2a). Step 1, we mapped the sequencing data to the pan-genome reference and identified an initial set of CNV bins based on the read depth with a window size of 100 Kb. Step 2, we applied a hidden Markov model to generate continuous CNV blocks (Additional file 2: Fig. S3), followed by the removal of short-length and low-frequency CNV blocks. CNV blocks sharing one border and have more than 80% overlapping regions were merged. CNVs closely linked in one cluster were further grouped. Then, an initial set of 8134 CNVb makers were identified genome-widely across the population. Step 3, we eliminated CNVb blocks with low recalls in ultra-low-coverage whole genome sequencing (ulcWGS) data (0.1×) to develop stable in silico markers (Additional file 2: Fig. S4). Finally, our pipeline yielded a total of 1240 non-redundant high-quality CNVb markers, comprising 1045 CNVb-deletion markers and 195 CNVb-duplication markers. By profiling these CNVb markers across the genome, we observed that these CNVb markers are distributed across all chromosomes, with an average of 59 markers per chromosome (Additional file 2: Fig. S5). We also showed that these CNVb markers span most regions of chromosomes, occupying up to 92.6% of each chromosome (Additional file 2: Fig. S6), indicating that the developed in silico DNA marker set offers the feasibility of representing wheat genomic variation genome-widely. We further performed saturation analysis of CNVb markers and showed that 95% of CNVb markers could be recalled when the panel size reached 230 (Fig. 2b), indicating the selected CNVb markers are sufficient for capturing the large CNV blocks at a population level in wheat.

### High recalls achieved by scanning CNVb markers in ultra-low-coverage sequencing data

To evaluate the performance of scanning the in silico CNVb markers in new varieties, we examined the recalls of CNVb markers by scanning sequencing datasets at various coverages, which were randomly sampled from high-coverage resequencing data. The results showed that the performance of developed CNVb markers exceeded raw CNV regions and SNPs, especially for the ultra-low coverage data. The CNVb markers achieved a mean recall of 99.3% even at coverage of 0.05× (Fig. 2c), highlighting the superiority of CNVb as a stable marker compared to traditional strategies. This result also suggests that CNVb could serve as a cost-efficient genotyping strategy for constructing DNA-based digitalized fingerprints of massive varieties based on ultra-low sequencing data.

### Linking CNVb markers with known structural variations and beneficial alleles

**Fig. 3:**
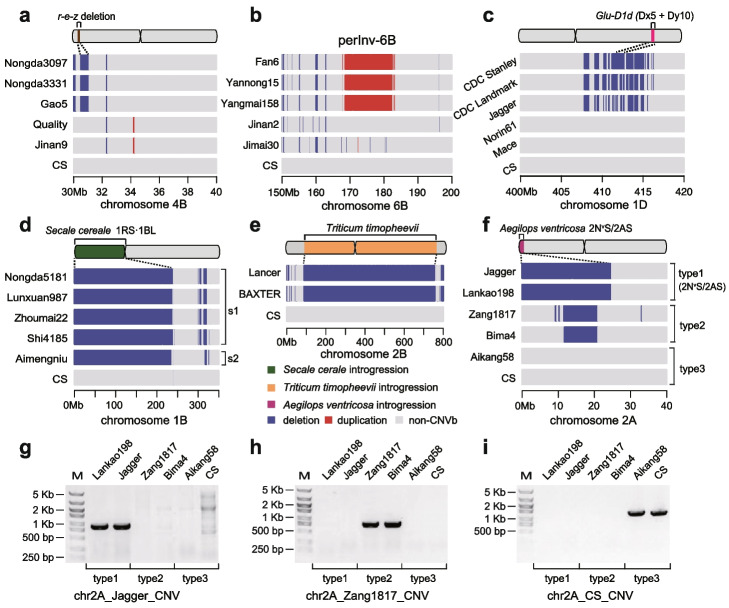
Associating CNVb markers with known structural variations and predominant haplotypes. **a** The CNVb-deletion marker CNVb.647 (chr4B: 30.5–31.1 Mb) corresponds to the *r-e-z* haplotype on chromosome 4B. **b** The CNV-duplication marker CNV.989 (chr6B: 167.9–183.4 Mb) is associated with the pericentric inversion on chromosome 6B (perInv-6B). **c** The CNVb-deletion marker CNV.162 (chr1D: 412.1–412.5 Mb) corresponds to the *Glu-D1d* (Dx5 + Dy10) allele of the high-molecular-weight glutenin gene *Glu-D1*. **d** The CNVb-deletion marker (CNV.67.1, chr1B: 0–239.3 Mb) associates with the 1RS·1BL translocation. The “s1” denoted the subtype 1RS·1BL translocation associated with CNVb.67.1 (chr1B: 0–239.3 Mb). The “s2” denoted the subtype 1RS∙7DL/7DS∙1BL translocation associated with CNVb.67.2 (chr1B: 0–236.7 Mb). **e** The CNVb-deletion marker CNVb.290 (chr2B: 89.5–769 Mb) corresponds to the introgression from *Triticum timopheevii* on chromosome 2B. **f** Distribution of three types of CNVb allelic genotypes within the first 40 Mb region of chromosome 2A. The first type of CNV allele (CNV. 189, chr2A: 0–24.7 Mb) corresponds to the introgressed segment from *Aegilops ventricosa*. **g**–**i** PCR validation on three types of CNVb alleles located in the 25 Mb telomeric region of chromosome 2A. M, DNA marker 5000. Two primers were designed using partial sequences of the introgression fragments in Jagger (**g**) and Zang1817 (**h**), respectively, and the other was designed using partial sequences from 0 to 24.7 Mb on chromosome 2A of the CS genome (**i**)

To fully harness the potential of wheat germplasm carrying beneficial alleles such as ones conferring disease resistance for breeding applications, we linked numerous well-known structural variations and beneficial alleles with CNVb markers (Table 1). The *r-e-z* haploblock on chromosome 4B with approximately 500 Kb deletion, characterized by simultaneous absence of the *Rht-B1*, *EamA-B*, and *ZnF-B* genes contributing to both the compactness and enhanced yield of semi-dwarf wheat [36], was digitized to a CNVb-deletion marker (CNVb.647, chr4B: 30.5–31.1 Mb) and identified in a total of 10 accessions (Fig. 3a, Additional file 1: Table S4). The pericentric inversion in chromosome 6B (perInv-6B), one of the most predominant chromosomal variants in wheat modern cultivars [21], was associated with a CNVb-duplication marker (CNVb.989, chr6B:167.9–183.4 Mb) on chromosome 6B. This association is based on the identification of a duplication block marker that is unique to varieties carrying the 6B inversion (Fig. 3b). Our study identified 12 previously reported accessions harboring perInv-6B [20, 21] and 21 additional accessions with the perInv-6B associated CNVb markers (Additional file 1: Table S5). The high-molecular-weight glutenin *Glu-D1d* (Dx5 + Dy10) allele is associated with superior bread-making quality [37]. We developed a CNVb-deletion marker (CNVb.162, chr1D: 412.1–412.5 Mb) corresponding to the *Glu-D1d* allele (Fig. 3c) and identified 11 accessions, such as Jagger, carrying the *Glu-D1d* allele (Additional file 1: Table S6), which was proven by SDS-PAGE [37, 38]. We identified 419 additional accessions that may carry the *Glu-D1d* allele (Additional file 1: Table S6). These intriguing results indicate the current panel of CNVb markers could serve as an alternative way for efficiently scanning the presence of beneficial alleles or key structure variations among wheat varieties.

**Table 1:** Information and genomic features of representative CNVb markers associated with known structural variations and predominant haplotypes

**Marker ID**	**Chromosome**	**Start position (Mb)**	**End position (Mb)**	**Associated genome feature**	**Associated gene**	**Number of lines**
CNVb.67.1	1B	0	239.3	1RS·1BL	*Pm8/Sr31/Lr26/Yr9*	111
CNVb.67.2	1B	0	236.7	1RS·7DL/7DS·1BL	*Pm8/Sr31/Lr26/Yr9*	16
CNVb.162	1D	412.1	412.5	*Glu-D1d* (Dx5 + Dy10) haplotype		430
CNVb.189	2A	0	24.7	2N^v^S/2AS	*Lr37/Yr17/Sr38*	142
CNVb.290	2B	89.5	769.0	*Triticum timopheevii* introgression	*Sr36*	2
CNVb.540	3D	592.0	616.0	*Thinopyrum ponticum* introgression		13
CNVb.647	4B	30.5	31.1	*r-e-z* deletion	*Rht-B1/EamA-B/ZnF-B*	10
CNVb.989	6B	167.9	183.4	perInv-6B		33

CNVb markers associated with reported interspecific introgressions in wheat were also annotated (Table 1, Additional file 1: Table S7). For instance, the well-documented 1RS introgression from rye (*Secale cereale*) carries multiple genes (*Pm8/Sr31/Lr26/Yr9*) contributing to disease resistance in wheat. In this study, we developed a CNVb-deletion marker (CNVb.67.1, chr1B: 0–239.3 Mb) associated with 1RS based on the high sequence divergence between the 1RS and 1BS (Fig. 3d). The CNVb.67.1 was identified in 16 accessions that were convinced as harboring the 1RS·1BL translocation by fluorescence in situ hybridization [20, 21], including Lunxuan987. Additionally, we reveal there are 95 more accessions that also have the 1RS·1BL translocation-associated CNVb markers (Additional file 1: Table S7). Our previous study showed the read depth pattern of 1B chromosome could well distinguish the two subtypes of 1RS-related translocation [16]. Based on the previous effort, we further developed a sub-CNVb marker on chromosome 1B (CNVb.67.2, chr1B: 0–236.7 Mb) in Aimengniu for representing the unique translocation (RT 1RS∙7DL/7DS∙1BL), which is different with the typical 1RS·1BL translocation in boundaries (Fig. 3d and Additional file 2: Fig. S7). Another example is the cultivar LongReach Lancer carrying two introgressed regions from different species, including a pericentric region spanning 427 Mb on chromosome 2B from *Triticum timopheevii* and a terminal segment of ~60 Mb on chromosome 3D from *Thinopyrum ponticum* [15]. Accordingly, the *Triticum timopheevii* related introgression is associated with CNVb markers CNVb.290 (chr2B: 89.5–769.0 Mb) (Fig. 3e), and the *Thinopyrum ponticum* related introgression is associated with CNVb markers CNVb.540 (chr3D: 592.2–616.0 Mb, Additional file 2: Fig. S8), respectively. By scanning our collection of wheat varieties, we showed the variety BAXTER also carries the *Triticum timopheevii* introgression (Fig. 3e). Collectively, we exemplified that structural variations and introgression haplotypes could be transformed into digitalized CNVb markers with application potential for scanning larger wheat variety panels.

As pervasive independent introgressions have been utilized in modern wheat breeding, we further showed CNVb marker could distinguish introgressions even with overlapped genomic coordinates. We identified two CNVb markers at the end of chromosome 2A short arm. The CNVb-deletion marker CNVb.189 (chr2A: 0–24.7 Mb) was detected in Jagger (Fig. 3f), which is linked to a 2N^v^S introgression from *Aegilops ventricosa* that conferred resistance to wheat blast and carried the rust disease resistance gene cluster (*Lr37/Yr17/Sr38*) [15]. An additional 141 varieties were detected with the CNVb.189 marker (Additional file 1: Table S8), such as Lankao198 (Fig. 3f). The second CNVb-deletion marker overlapped with CNVb.189 is CNVb.173 (chr2A: 11.5–21.0 Mb), which was detected in Tibetan semi-wild wheat accession Zang1817 and Chinese cultivar Bima4 (Fig. 3f). Collinearity analysis between Zang1817 and CS genome showed a degree of collinearity in the deletion region, despite low-quality alignment (Additional file 2: Fig. S9), indicating that the CNVb.173 marker corresponds to an interspecific introgression. 50.9% of wheat varieties showed no CNVb maker detected in the first 25 Mbp regions of chromosome 2A, indicating three types of alleles as distinguished by CNVb markers. To validate the three identified alleles, we performed a PCR analysis by designing primers specific to 2N^v^S introgression sequences in the Jagger assembly (Fig. 3g), to the sequences in the Zang1817 assembly (Fig. 3h), and to the wild-type Chinese Spring assembly (Fig. 3i), and results showed the yielded amplification in Jagger, Lankao198, Zang1817, Bima4, CS, and Aikang58 matched with the predicted allele types by CNVb markers. This experiment validated the accuracy of the CNVb marker and proved the authenticity of the CNVb marker-based strategy in distinguishing multiple allele types, even without fully assembled sequences, saving effort compared to traditional SNP/InDel/SSR markers.

### Digital fingerprinting map of wheat varieties utilizing CNVb markers

**Fig. 4:**
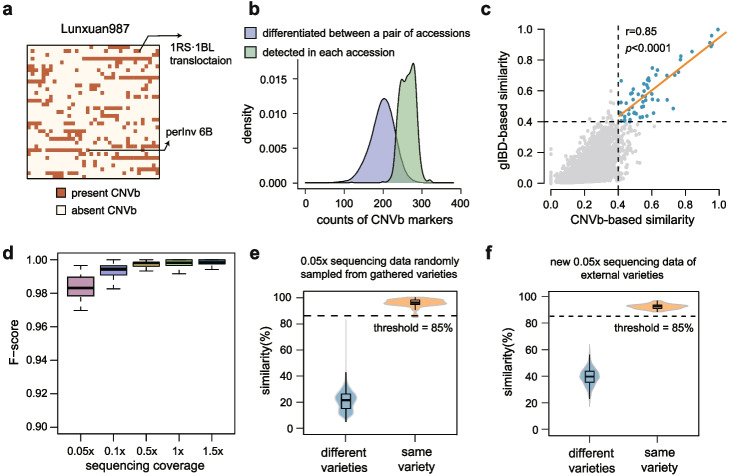
Performance evaluation of CNVb markers in germplasm identification. **a** The CNVb marker fingerprint of Lunxuan987. CNVb marker fingerprint consists of a QR-code-like two-dimensional matrix, with each cell representing a CNVb marker. All the markers are ordered by chromosomes and are filled into the matrix by rows, from left to right and from top to bottom. Two specific markers were highlighted by arrows with annotated descriptions as interspecific introgression or structural variation. **b** Spectrum of the number of CNVb markers in each accession and the number of differential CNVb markers in pairwise accessions. **c** Variety similarity was calculated based on CNVb markers and germplasm resource-based Identity-By-Descent (gIBD) block, respectively. Varieties with similarities calculated by both methods above 0.4 are highlighted in blue in the upper right corner. The upper right corner also displays the regression trend between a variety of similarities calculated based on CNVb markers and gIBD. Vertical and horizontal dashed lines represent variety similarity equal to 0.4, respectively. *r*, Pearson’s correlation coefficient, *P* value < 2.2 × 10^−12^. **d** The accuracy of identifying CNVb fingerprints in accessions at low sequencing coverage. The ulcWGS data are simulated from whole genome sequencing data of 100 accessions randomly selected from the original CNVb marker library construction. **e** The similarity of pairwise accessions from two batches of 0.05× simulated sequencing datasets, each comprising 100 randomly selected accessions from our dataset. The dashed line represents the threshold (85%) for variety identification. **f** The similarity of pairwise accessions from two batches of 0.05× sequencing data, each containing 100 accessions not included in the original CNVb marker library. The dashed line represents the similarity threshold (85%) for variety identification

We constructed a comprehensive CNVb fingerprint map consisting of 1599 accessions, by further integrating public resequencing data of 1071 wheat accessions [39–41] (Additional file 1: Table S1). Moreover, we created a QR-code-like two-dimensional CNVb markers profile for each accession (Fig. 4a). The presence of a CNVb-duplication or a CNVb-deletion marker in a variety indicates that this variety contains the duplication or deletion block, respectively. For instance, the profile of Lunxuan987 showed that 276 CNVb markers were detected as present, including the 1RS·1BL translocation marker and perInv-6B marker. In the CNVb fingerprint map, the number of CNVb markers present in each accession ranges from 119 to 322. The genotypes of 199 markers are different between pairwise accessions on average (Fig. 4b), and there are more than 100 markers with different genotypes for 99.5% of the accession pairs (Fig. 4b). For example, the sibling cultivars Bima1 and Bima4 present 117 distinct markers (Additional file 2: Fig. S10). These results indicate a great potential of CNVb markers for discriminating closely related accessions. To further evaluate the accuracy of variety discrimination using CNVb markers, we compared CNVb strategy and germplasm resource-based Identity-By-Descent (gIBD), a previous strategy evaluating the genome-wide similarity that could reflect pedigree relationships in various plant species [16]. The results showed that the similarity estimated by the CNVb-based strategy is highly correlated with gIBD-based similarity, and the correlation is especially significant for varieties with a close genetic relationship (similarity > 0.4, Pearson’s correlation = 0.85, *P* < 2.2 × 10^−12^) (Fig. 4c). Thus, the result demonstrated the CNVb markers can serve as an effective strategy for the reliable estimation of genetic similarity to distinguish the genetic-similar wheat accessions.

### Boost germplasm identification with ultra-low-coverage sequencing

Genetic identification of germplasm resources is crucial for protecting breeders’ rights and promoting its digital management. Reliability tests of CNVb markers among various depths (0.05×, 0.1×, 0.5×, 1.0×, and 1.5×) showed that the minimum recall and precision ratio observed at these reduced depths were above 99.0% and 97.9%, respectively, for each accession (Fig. 4d), demonstrating the robustness of CNVb fingerprints in ulcWGS. To further examine the power of CNVb fingerprints in distinguishing germplasm under ulcWGS, we selected 100 accessions from the original CNVb marker library and compared the CNVb fingerprints estimated at both the original and downsampled sequencing coverages. The similarity between pairwise accessions exhibited a bimodal distribution with two distinct peaks, which corresponded to the similarity between the same varieties and between different varieties (Additional file 2: Fig. S11). A similarity of 85% was selected as the threshold for variety differentiation based on the 99% confidence interval of the “distinct variety” distribution to ensure high accuracy in distinguishing varieties. The results showed that more than 99.9% of varieties could be accurately classified when the sequencing depth surpassed 0.05× (Fig. 4e, Additional file 2: Fig. S12), verifying the CNVb fingerprint-based germplasm identification strategy at ultra-low sequencing coverage. To assess the generalization ability of this strategy, 100 accessions not among the original accessions used to construct the CNVb marker library were randomly selected and subjected to two rounds of downsampling to 0.05× ulcWGS data, creating two replicate datasets. Pairwise comparisons of these accessions confirmed that the strategy with a threshold of 85% can effectively differentiate accessions, as well as replications of the same accessions (Fig. 4f). This demonstrates the practicality and accuracy of CNVb markers in ulcWGS for germplasm identification.

### WheatCNVb database for exploring and comparing CNVb profiles

**Fig. 5:**
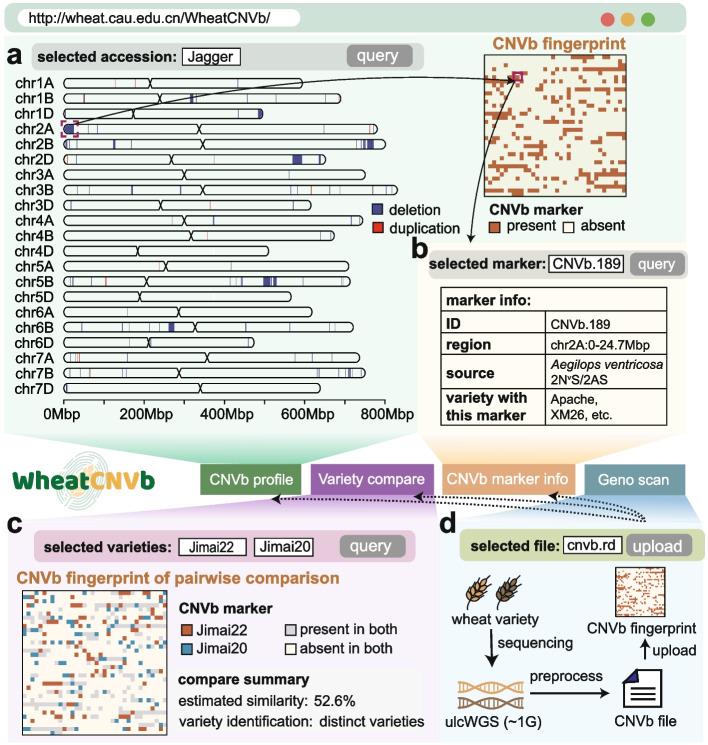
Schematic of the WheatCNVb database. **a** The “CNVb profile” presents an example of the distribution of the CNVb marker and the CNVb fingerprint barcode of Jagger. **b** The “CNVb marker info” function provides a table including marker ID, location, introgression source, and relevant accessions of each CNVb marker. **c** The “Variety compare” function shows the CNVb fingerprints in the mode of pairwise comparison, also with the estimated similarity. **d** The “Geno scan” function allows users to upload a bin-wised read-depth profile, which can be calculated with ultra-low whole genome sequencing data, and generate accession-specific CNVb fingerprint for variety identification and similarity evaluation

To enhance the accessibility of CNVb markers, we developed a database named WheatCNVb (http://wheat.cau.edu.cn/WheatCNVb/), based on the profiling of 1599 hexaploid wheat accessions with 1240 CNVb markers. Generally, the WheatCNVb database offers four main functions. First, the “CNVb profile” function allows users to query the CNVb profile for each accession. Two visualization modes were offered, as a chromosomal profile with colored regions representing the presented markers, and a QR-code-like representation of the digital present-absent status of 1240 CNVb markers (Fig. 5a). Second, the “CNVb marker info” function provides detailed information on CNVb marker, including the marker ID, location, annotations, and the accession list that harbors this marker (Fig. 5b). Third, the “Variety compare” function supports the comparison of CNVb fingerprints for any selected pair of accessions, which can intuitively visualize the shared and differential CNVb markers and estimate the similarity based on the CNVb profiles (Fig. 5c). For example, a pairwise analysis using the WheatCNVb database revealed a 52.6% genetic similarity between Jimai22 and Jimai20 (Fig. 5c). Additionally, each variety possesses 90 and 80 unique CNVb markers, respectively (Fig. 5c), confirming their classification as distinct wheat varieties.

Moreover, the “Geno scan” function enables users to analyze customized wheat accessions. Users can perform an ulcWGS to their material, locally prepare the bin-wised read depth file of the accession locally with a pipeline provided on the webpage (http://wheat.cau.edu.cn/WheatCNVb/tutorial.html), and upload the file to the WheatCNVb database (Fig. 5d). The database will facilitate the identification of CNVb markers for the accession, obtaining a CNVb fingerprint that can be compared with varieties stored in the database or other submitted varieties for comprehensive variety identification.

## Discussion

High-throughput, affordable, and rapid detection of DNA-based markers is essential for exploring germplasm diversity and protecting breeders’ rights. However, developing an efficient genotyping tool for crops like wheat, with its huge and complex genome, remains challenging. Despite the abundance of SVs (including CNVs), which are crucial polymorphisms in crops, efforts to develop automated platforms for CNV typing are limited [13]. In this study, we revealed that the high frequency and polymorphism of large CNV blocks in wheat make CNVb an effective DNA-based marker for efficient variety identification. We generated a comprehensive reference catalog of CNV blocks at the pan-genome level, which captures sequence polymorphisms absent in Chinese Spring and provides sufficient CNVb markers to perform accurate variety identification. Additionally, we addressed the high rates of false positives and negatives in CNVb calling specific to ulcWGS by refining and merging raw CNV blocks. We manually annotated the tagged CNVb markers with known structural variations and beneficial alleles, and we developed an ulcWGS scanning strategy for new candidate varieties, which demonstrated advanced performance in germplasm identification.

Genotyping gene bank collections is a crucial first step in harnessing the untapped biodiversity of wheat genetic resources [42]. To date, over 560,000 wheat accessions are preserved in near 40 gene banks worldwide [11]. Despite significant progress in collecting wheat resources, the capacity to identify, integrate, and utilize such extensive germplasm remains markedly insufficient [3, 43]. Compared to conventional methods for assessing wheat genetic resources in gene banks, CNVb markers showed multiple aspects of advantages (Additional file 2: Table S9). First, CNVb markers significantly reduced the cost for genotyping per marker compared to Southern blot-based markers like RFLP and chip-based markers like SNP arrays, while being comparable in cost-effectiveness to SSRs and GBS. Second, CNVb markers support ultra-low-depth high-throughput sequencing and can be fully automated, which is more labor-saving and less equipment-dependent than widely used SSR markers, making them more suitable for large-scale applications. Third, CNVb markers provide very high reliability, comparable to SNP arrays, and better performance than the GBS strategy. Fourth, CNVb markers provide high accuracy in variety identification, capable of distinguishing even closely related accessions (>40% similarity), comparable to genome-wide gIBD analysis using high-coverage whole genome sequencing (Fig. 4c). Fifth, CNVb markers support capturing larger genomic variations, which provides unique genetic information and is crucial for identifying traits linked to structural variations. This feature is particularly advantageous in polyploid crops like wheat, where large genomic structural variations are prevalent. Thus, CNVb markers represent a low-cost, high-throughput, labor-saving, and highly reliable tool for modern breeding and germplasm management. The current plant variety protection system relies on phenotype-based distinctness, uniformity, and stability assessments, which can be costly, time-consuming, and often limited to a small number of traits influenced by environmental conditions [8]. Moreover, with the emergence of new breeding technologies that facilitate minor modifications in varieties, yielding specific merits or utilities, the challenge of detecting distinctness between varieties, especially those that are essentially derived, is increasing [44, 45]. CNVb marker is cost-efficient, high throughput, and highly accurate, making it a practical alternative to morphological trait and traditional molecular markers. It provides a low-cost, thousand-marker one-time, and rapid technical solution, ideal for establishing an evaluation system for essentially derived varieties.

The initial hybridization of bread wheat involved a limited number of individuals, where the diploid *Aegilops tauschii* (DD) was hybridized with the tetraploid *Triticum turgidum* (AABB) to form the allohexaploid *Triticum aestivum* (AABBDD), resulting in lower genetic diversity compared to its progenitors [46]. To address this, farmers and early breeders incorporated members from secondary and tertiary gene pools into wheat breeding programs [17, 19]. However, the absence of a high-throughput, cost-effective, and precise identification strategy hinders the resolution and utilization of numerous SVs and interspecific introgressions within the wheat genome. Our PCR analysis suggests that CNVb markers can be associated with various types of genomic variations, indicating their potential as effective signals for tracking documented SVs and introgression events.

This study serves as a preliminary exploration for the development of wheat CNVb markers. Our findings suggest that CNVb is the optimal choice for identifying large SVs and introgression within the wheat genome, as well as for variety identification. Considering the current limited availability of resequencing data, and the abundance of whole exome sequencing and microarray data, our future efforts will focus on integrating these data sets to update and expand the CNVb marker collection, which will facilitate the discovery of rare CNVbs. Additionally, associating these markers with phenotype data will help in nominating key CNVb markers to assist in the wheat breeding programs. The CNVb marker identification strategy outlined in this study also shows promise for application in other crops.

## Conclusions

Our study introduces a CNVb-based genotyping approach that could enhance the digitalization and management of wheat germplasm resources using ultra-low-coverage sequencing. The CNVb markers, validated by PCR analysis, not only facilitate the discrimination of massive wheat varieties but also link key genetic traits and beneficial alleles. The WheatCNVb platform further supports this approach by providing a dynamic, user-friendly interface for the exploration and comparison of CNVb profiles, embodying a practical tool for breeders and researchers. Overall, the CNVb-based approach promises a low-cost and high-throughput genotyping strategy for enabling digitalized wheat germplasm management and modern breeding with precise and practical decision-making.

## Methods

### Collection and variation calling of wheat resequencing data

A total of 1599 published wheat accessions with whole genome resequencing data [16, 17, 25–27, 39–41] (Additional file 1: Table S1) were used in this study. Trimming of raw reads was performed using Trimmomatic, followed by the mapping of high-quality reads to the wheat pan-genome via BWA-MEM [47]. Bamtools v2.4 [48] was used to filter read pairs with either abnormal insert sizes (>10,000 bp or =0 bp) or low mapping quality scores (<1). Samtools v1.3 [49] was then employed to remove any potential PCR duplicate reads.

### Construct non-Chinese Spring chromosome at pan-genome level (chrNCP)

To construct a wheat pan-genome, we first collected de novo assembled genomes of 17 wheat varieties [15, 26, 28, 31–35], including the reference assembly of Chinese Spring RefSeq v1 (CS). Excluding CS, the remaining 16 genomes were ranked based on contig N50 length and whether Hi-C sequencing was used for scaffolding (Additional file 1: Table S2). We identified absent sequences in the CS genome from the 16 varieties using a whole-genome iterative alignment strategy. The alignment process involved trimming raw reads using Trimmomatic, followed by mapping high-quality reads to the wheat pan-genome with BWA-MEM [47]. Bamtools v2.4 [48] was used to filter read pairs with abnormal insert sizes (>10,000 bp or =0 bp) or low mapping quality scores (<1). Samtools v1.3 [49] was employed to remove potential PCR duplicate reads. Starting with the highest-ranked genome Aikang58 genome as the reference, we aligned CS resequencing data to Aikang58, using a 1 Mb sliding window and a read-depth based method to detect sequences absent in CS relative to Aikang58. This procedure was iteratively applied, comparing CS and Aikang58 resequencing data against the second-ranked Fielder genome to identify non-redundant deletion blocks relative to Fielder, and continued through all 16 varieties. Through this methodology, we extracted non-redundant deletion block sequences absent in CS, which were assembled in chromosomal order into “chrNCP” as a supplementary genome sequence to the CS reference. Thus, “chrNCP” combined with the CS genome forms the wheat pan-genome (Additional file 1: Table S4).

### Identification of CNV blocks

The genome was segmented into 100 Kb nonoverlapping windows to calculate the average read depth, utilizing the “coverage” function in bedtools v2.27.1 [50]. These counts were then normalized by dividing them by the mode of the read depth across the genome. According to the distribution pattern of normalized read counts, which showed a near-normal distribution centered around a value of 1, windows exhibiting normalized read counts below 0.5 or above 1.5 were classified as deletion and duplication windows, respectively. Finally, contiguous deletion and duplication windows were merged to delineate whole-genome CNV blocks.

### Development of CNVb markers

To develop CNVb markers from 528 resequenced varieties, the identification of raw CNV blocks was refined through a systematic process structured into three main steps.

Step 1: Filtering of raw CNV blocks. Initially, for CNV blocks aligned to the CS reference, we employed a multinomial hidden Markov model (HMM) using the hmmlearn Python library (https://pypi.org/project/hmmlearn/) to minimize random noise and enhance the clarity of CNV block patterns. This model was configured with parameters set to “n_components=3, n_iter=60, tol=0.001” and optimized via the Baum-Welch iterative re-estimation algorithm through the “fit()” method. The “decode()” method, with “algorithm=viterbi”, was then used to smooth and decode CNV blocks. CNV blocks with a value of (length / 100 Kb + N) ≤ 10 were further filtered out, where “N” indicates the number of accessions containing the CNV block and “length” indicates the length of CNV block. For CNV blocks mapped to the “chrNCP” genome, a similar filtration and refinement were applied, excluding CNV blocks with a value of (length / 1 Mb + N) ≤ 10 or (length / 1 Mb + n) ≤ 10, where “n” indicates the number of accessions without the CNV block.

Step 2: Merging CNV blocks. For CNV blocks within the CS reference regions, redundancy was addressed by merging significantly overlapping blocks (*ρ*_*o*_ ≥ 0.8) and merging linked blocks (those within 5 Mb apart and with *ρ*_*link*_ ≥ 0.9). The formulas for *ρ*_*o*_ and *ρ*_*link*_ are defined as:

$${\rho }_{o}=\frac{{L}_{o}}{{L}_{1}+{L}_{2}-{L}_{o}}$$

$${\rho }_{link}=\frac{{C}_{s}}{{C}_{1}+{C}_{2}-{C}_{s}}$$

where *L*_1_ and *L*_2_ are the lengths of the CNV blocks, *L*_*o*_ is the overlapping length, *C*_1_ and *C*_2_ are the counts of accessions carrying each CNV block, and *C*_*s*_ is the count of accessions with both CNV blocks. No further processing was needed for already filtered CNV blocks corresponding to the “chrNCP” genome. This merging step resulted in a preliminary CNV marker library, encompassing multiple CNV blocks per marker. Markers identified in both the CS reference and “chrNCP” sequences were assessed for redundancy with a specific focus on their presence or absence across accessions. If the genotype of a marker form “chrNCP” is highly correlated with that of another marker from CS, the marker from the “chrNCP” sequence will be filtered out.

Step 3: Filtering CNVb markers for ulcWGS stability. To ensure the applicability of CNVb markers for ulcWGS data, markers indistinguishable at low sequencing coverage were excluded. CNV blocks were initially genotyped from hcWGS and simulated 0.1× coverage data, with the latter obtained by downsampling hcWGS data. Each accession’s CNV blocks were compared with the preliminary marker library to ascertain the presence or absence of CNVb markers in both hcWGS and 0.1× coverage data. A marker was considered present if at least one CNV block overlapped with it by ≥90% and the length discrepancy between the CNV block and the marker is less than 1 Mb. Markers with inconsistent detections in more than 10 accessions were removed. The refined set of CNVb markers formed the finalized marker collection.

### Construction of the low-coverage sequencing test set

To create a test set for ulcWGS, 100 accessions with sequencing depths > 5× were randomly selected (Additional file 1: Table S5). Their original BAM files were downsampled to depth levels of 0.01×, 0.05×, 0.1×, 0.5×, 1×, and 1.5×, thereby generating simulated ulcWGS data using Samtools v1.3.1 [49]. CNV blocks were then genotyped for each accession’s ulcWGS data. These identified CNV blocks from each accession were compared with the raw CNVb marker library to ascertain the presence or absence of each CNVb marker in the simulated ulcWGS data.

### Identification of CNVb markers using ulcWGS

The pipeline is to first identify the type of CNV blocks and then match these CNV blocks to the corresponding markers to identify which markers are present in each variety (Additional file 1: Fig. S4). Initially, the ulcWGS data are aligned to the pan-genome to detect raw deletion (copy number = 0) and duplication (copy number ≥ 2) blocks. These blocks are then separately compared with their corresponding CNVb marker set. The presence of a deletion or duplication marker in a variety is determined based on the following criteria, if a deletion or duplication block present in the variety overlaps with a deletion or duplication marker by at least 90% and the difference in length between the block and the marker is less than 100 Kb.

### Evaluating lcWGS recall for SNPs, raw CNVb, and CNVb markers

SNPs were detected in all 100 accessions using GATK v3.868’s HaplotypeCaller module in GVCF mode. To assess the recall rates for SNPs, raw CNVb, and CNVb markers identified via low-coverage sequencing, these findings were benchmarked against results from high-coverage sequencing.

### PCR analysis

The primer sequences for three marker types were designed based on distinct introgression fragments. Type 1 marker primers were derived from an introgression fragment in the Jagger genome, corresponding to the CNVb-deletion type 1 (CNVb.139, chr2A: 0–24.7 Mb). The forward primer was 5′-TGCATGTCACTACCACGACC-3′, and the reverse primer was 5′-ACAACCCGTTTTCTTCACGG-3′. Type 2 marker primers were selected from an introgression fragment in the Zang1817 genome, corresponding to the CNVb-deletion type 2 (CNVb.142, chr2A: 12.0–21.3 Mb). The forward primer was 5′-TACTTTCGGATTGACAATTATCCTCTTATC-3′, and the reverse primer was 5′-TGGAAAAATGGTCTTACGGTTATATGAAAT-3′. For the type 3 marker, primers were selected from a segment of the CS genome sequence, aligning with the region of CNVb-deletion type 2 (CNVb.142, chr2A: 12.0–21.3 Mb). The forward primer was 5′-GAACTGATTACAAATGAATAGTTGTAGGGA-3′, and the reverse primer was 5′-TTAGTTACACCATGAGTTAGCATCATTTAG-3′. The PCR reaction system was 20 μL, including 10 μL 2× M5 HiPer plus Taq HiFi PCR mix, 1 μL forward primers and 1 μL reverse primers (10 μmol L^−1^), 2 μL template DNA (150 ng μL^−1^), supplemented with ddH_2_O to 20 μL. The PCR conditions were 95 °C for 5 min, followed by 35 cycles of 95 °C for 30 s, 60 °C for 30 s, and 72 °C for 5 min, and finally followed by 72 °C for 5 min.

### Calculation of the pairwise similarity

The pairwise similarity between accessions was calculated based on their CNVb fingerprints. The formula for similarity is defined as:

$$similarity=\frac{{M}_{share}}{{M}_{s1}+{M}_{s2}-{M}_{share}}$$

where *M*_*S*1_ and *M*_*S*2_ represent the number of markers in the first and second accessions, respectively, and *M*_*share*_ denotes the number of markers shared between the two accessions.

### Assessing the accuracy of variety identification based on ulcWGS

This study evaluates the accuracy of variety identification using ulcWGS by comparing it with hcWGS. The test set of 100 accessions from ulcWGS is designated as replicate 1, while an identical set of 100 accessions sequenced at a high depth forms replicate 2. CNVb fingerprints are used for pairwise comparisons between the replicates to simulate the variety identification process. The similarity between each pair is calculated, with a threshold of 85% similarity set to determine if the accessions are of the same or different varieties. Power of variety identification is defined as the proportion of correctly identified distinct variety pairs out of the total distinct pairs.

In addition, we randomly selected 100 accessions that were not among accessions used to construct the CNVb marker library and performed downsampling on their original BAM files to 0.05× coverage in two separate batches using Samtools v1.3.1 [49]. This process generated two sets of 0.05× simulated sequencing data. The CNVb fingerprints from both data sets were then subject to pairwise comparisons, designating the first data set as replicate 1 and the second as replicate 2. We calculated the similarity between the two replicates, setting a similarity threshold of 85% for variety identification. The statistical power (1 − β) was also computed as the standard for evaluating the accuracy of varietal identification.

## Supplementary Information

Additional file1 (XLSX 233 KB)

Additional file2 (PDF 2597 KB)

Additional file3 (DOCX 1516 KB)

## Data Availability

The raw reads of 1599 previously published resequenced accessions are available under the following NCBI Sequence Read Archive accessions: PRJNA544491 [15], PRJNA722149 [16], PRJNA476679 [17], PRJNA597250 [25], PRJNA596843 [26], PRJNA439156, PRJNA663409 [27], PRJEB48988, PRJEB48738 [39], and the National Genomics Data Center (https://bigd.big.ac.cn/gwh) database under project CRA005878 [40]. The previously published de novo assembled reference genome sequences are available under the following NCBI Sequence Read Archive accessions: PRJNA544491, PRJEB37938, PRJNA492239, PRJNA528431, PRJEB39558, PRJEB35709 [15], PRJNA595806 [26], PRJEB44721 [32], PRJEB45541 [33], and PRJEB49351 [34]. They are also available in the National Genomics Data Center (https://bigd.big.ac.cn/gwh) database under accession number GWHANRF00000000 [31] and the BIG Data Center (https://bigd.big.ac.cn/) under BioProject numbers PRJCA004332 [35]. The database WheatCNVb is available as open-source code under the MIT License at https://github.com/Niujx98/WheatCNVbDB [51] and the raw data can be accessed on Zenodo (10.5281/zenodo.11403154) [52]. The CNVb detection pipeline and corresponding manual are available as open-source code under the MIT License at https://github.com/Niujx98/WheatCNVbScan [53] and can be accessed on Zenodo (10.5281/zenodo.11401875) [54].
