# Publisher Correction: Tagging large CNV blocks in wheat boosts digitalization of germplasm resources by ultra-low-coverage sequencing

**DOI:** 10.1186/s13059-024-03442-0

**Published:** 2024-11-25

**Authors:** Jianxia Niu, Wenxi Wang, Zihao Wang, Zhe Chen, Xiaoyu Zhang, Zhen Qin, Lingfeng Miao, Zhengzhao Yang, Chaojie Xie, Mingming Xin, Huiru Peng, Yingyin Yao, Jie Liu, Zhongfu Ni, Qixin Sun, Weilong Guo

**Affiliations:** 1 Frontiers Science Center for Molecular Design Breeding, Key Laboratory of Crop Heterosis and Utilization, Beijing Key Laboratory of Crop Genetic Improvement, China Agricultural University, Beijing, 100193 China; 2 Sanya Institute of China Agricultural University, Sanya, 572025 China

**Correction** **:** **Genome Biol 25, 171 (2024)**

**https://doi.org/10.1186/s13059-024-03315-6**

Following publication of the original article [1], the authors identified a typesetting error, whereby the equal contribution statement was mistakenly omitted. The correct statement is as follow: Jianxia Niu, Wenxi Wang and Zihao Wang are co-first authors and contributed equally.

The original article [1] has been corrected.
