# Supplementary material for: Tagging large CNV blocks in wheat boosts digitalization of germplasm resources by ultra-low-coverage sequencing
Source: Genome Biol. 2024 Jul 1;25:171. doi: 10.1186/s13059-024-03315-6 (PMC11218387; doi:10.1186/s13059-024-03315-6)
Supplement: Supplementary file 2 — Additional file2 (PDF 2597 KB) [file 13059_2024_3315_MOESM2_ESM.pdf]

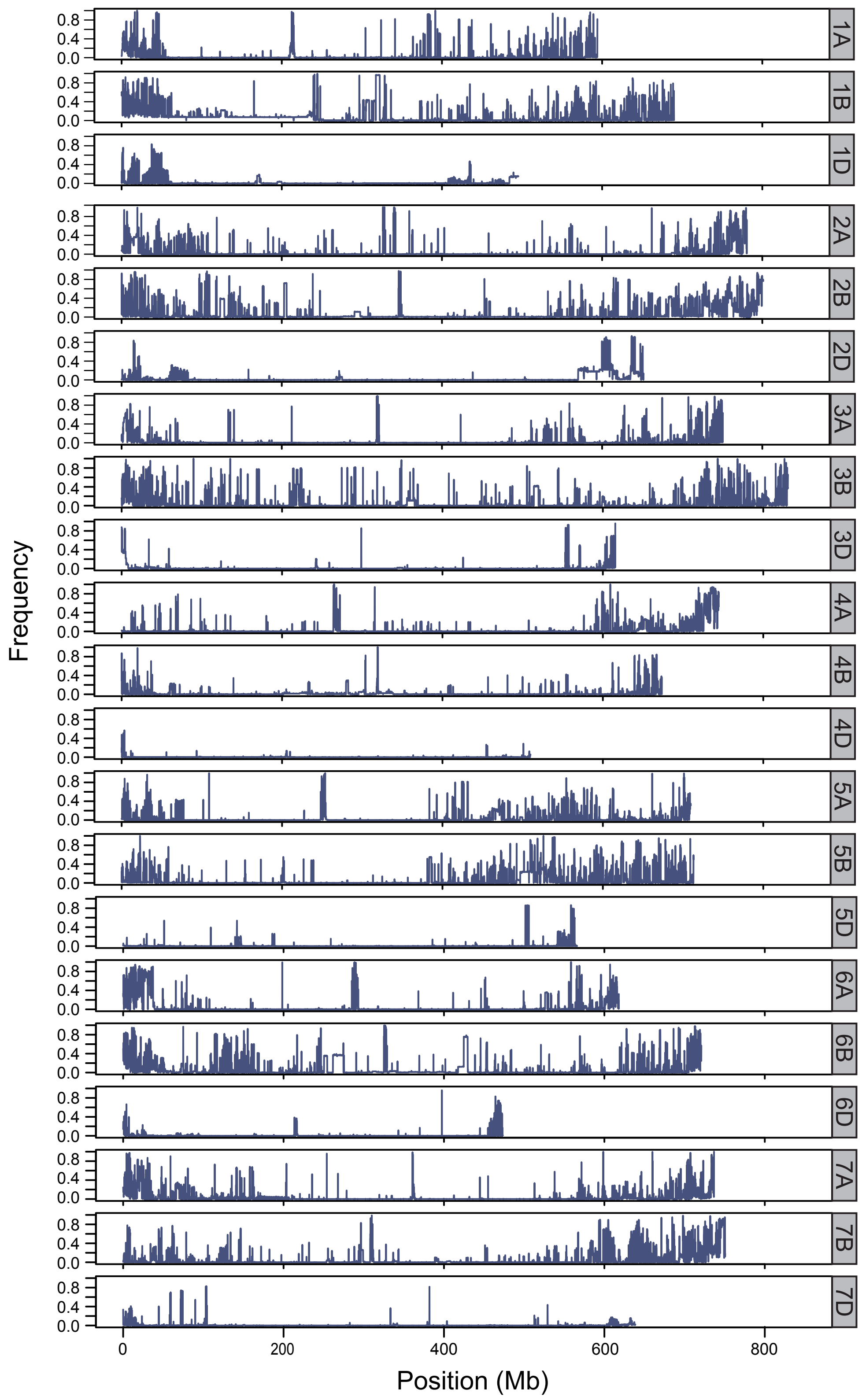

Fig S1

CNV distribution across the chromosomes in wheat. Bin size, 100 Kb.

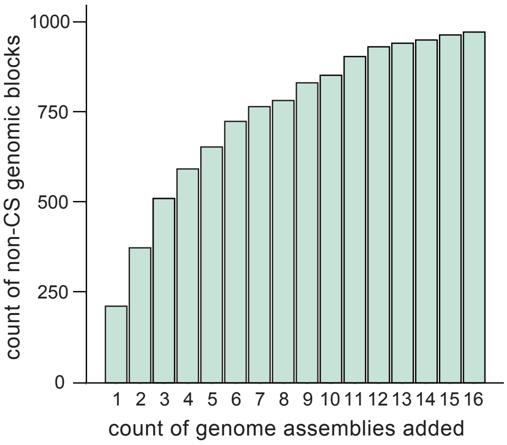

Fig S2

The cumulative count of unique non-CS genomic blocks identified during the construction of a pan-genome with the sequential addition of genome assemblies.

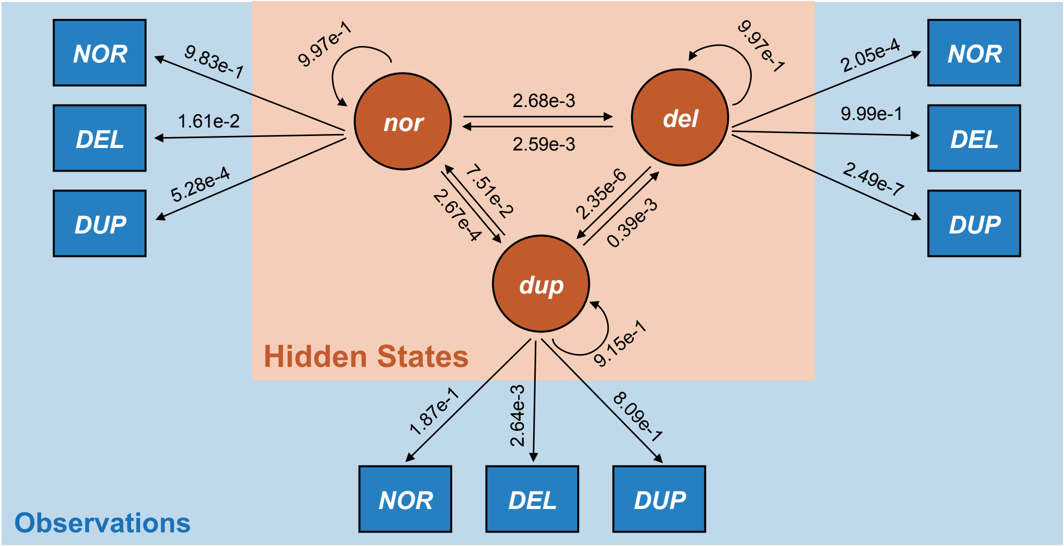

Fig S3

Layout of the hidden Markov model (HMM) for filtering CNVb markers. The genome windows are classified into three classes: normal (NOR) with no CNV appearing, deletion (DEL), and duplication (DUP) during the identification of CNV blocks. The sequence of CNV status along the genome was regarded as the observations of the HMM (blue boxes in the figure). The hidden states (orange circles, “nor” for no CNV appearing, “del” for deletion, and “dup” for duplication) are regarded as the real CNV status of each genome window, which may be observed as different statuses at a specific probability (marked near the arrow from the orange circles to blue boxes). Following the hypothesis of the Markov chain, the probability of “real” CNV status transiting from one status to another was marked near the arrow between orange circles.

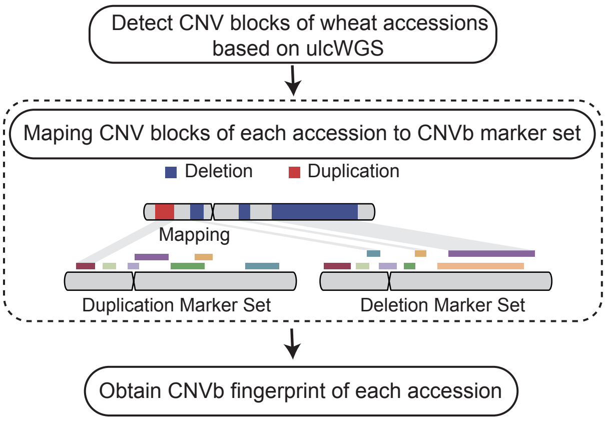

Fig S4

Workflow for CNVb marker scanning in wheat varieties based on ulcWGS. The process begins with the detection of CNV blocks in wheat accessions utilizing ulcWGS. Subsequently, identified CNV blocks of each accession are mapped against the CNVb marker library. If a CNVb shows significant overlap with a CNVb marker, the wheat accession is considered to carry that specific CNVb marker. The collective mapping of these CNV blocks to the CNVb markers enables the establishment of a CNVb fingerprint for each accession, which is a composite representation of the presence or absence of specific CNVb markers in the genome.

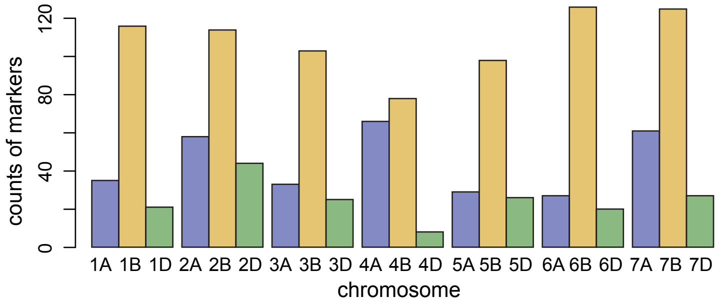

Fig S5

Count of CNVb markers on each chromosome.

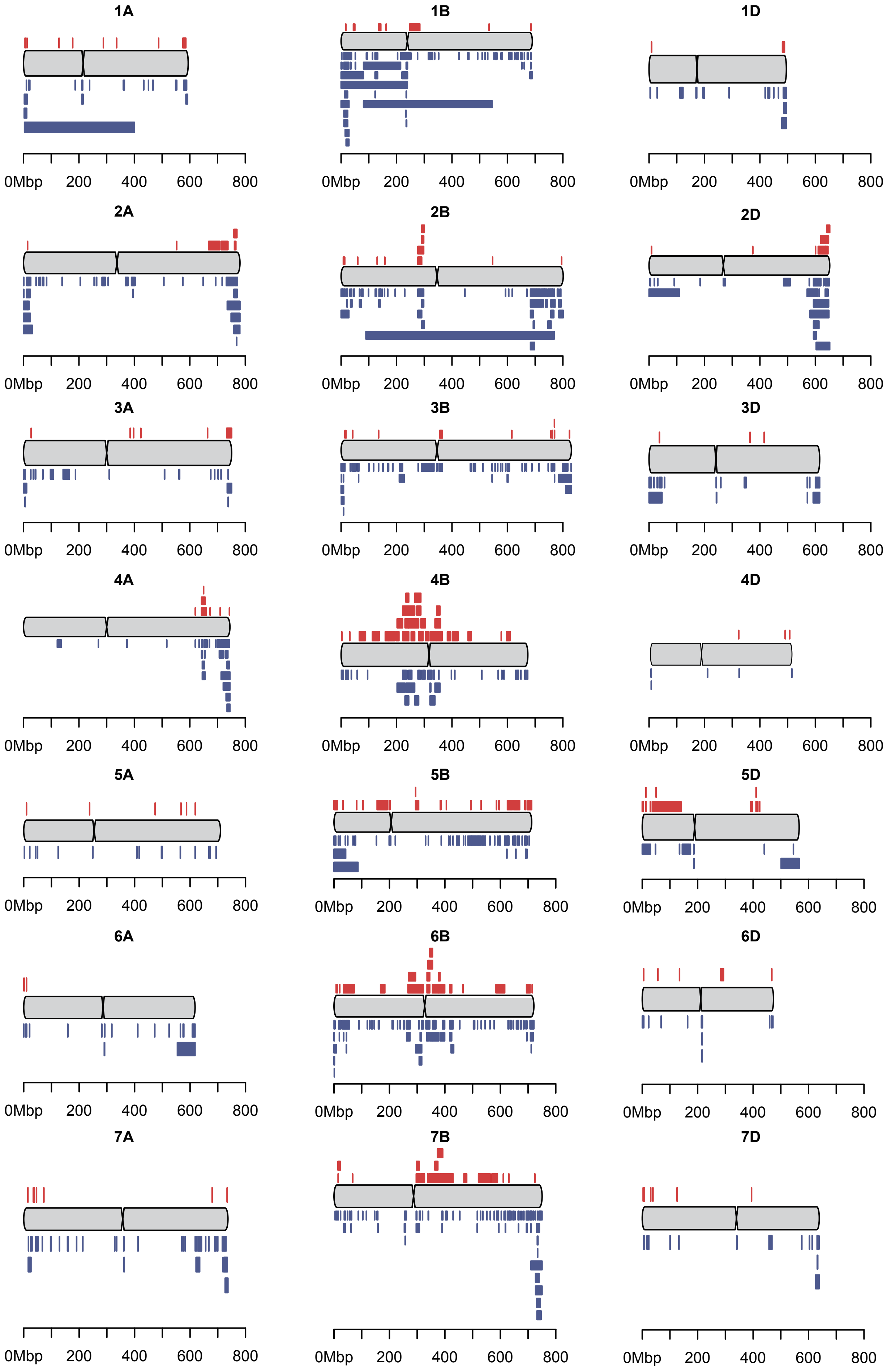

Fig S6

Distribution of CNVb markers across each chromosome. Blue blocks represent deletion block markers, while red blocks indicate duplication block markers. Stacked blocks denote distinct CNVb markers with overlapping regions.

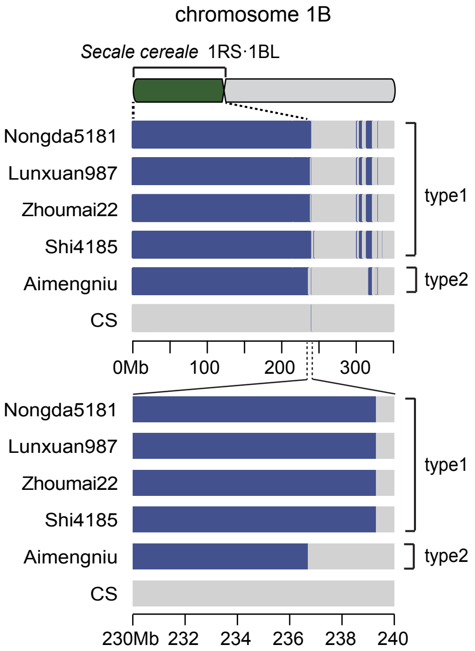

Fig S7

The CNVb-deletion marker associates with the 1RS·1BL translocation. The “type 1” denoted the subtype 1RS·1BL translocation associated with CNVb.67.1 (chr1B: 0-239.3 Mb). The “type 2” denoted the subtype 1RS·7DL/7DS·1BL translocation associated with CNVb.67.2 (chr1B:0-236.7 Mb).

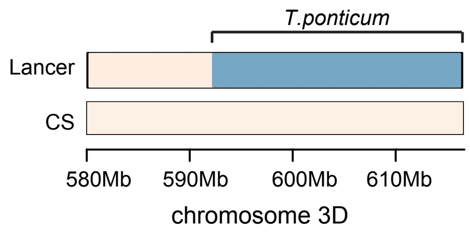

Fig S8

The CNVb.541 CNVb-deletion marker (chr3D: 592.1-616.0 Mb) corresponds to the introgression from *Thinopyrum ponticum* on chromosome 3D.

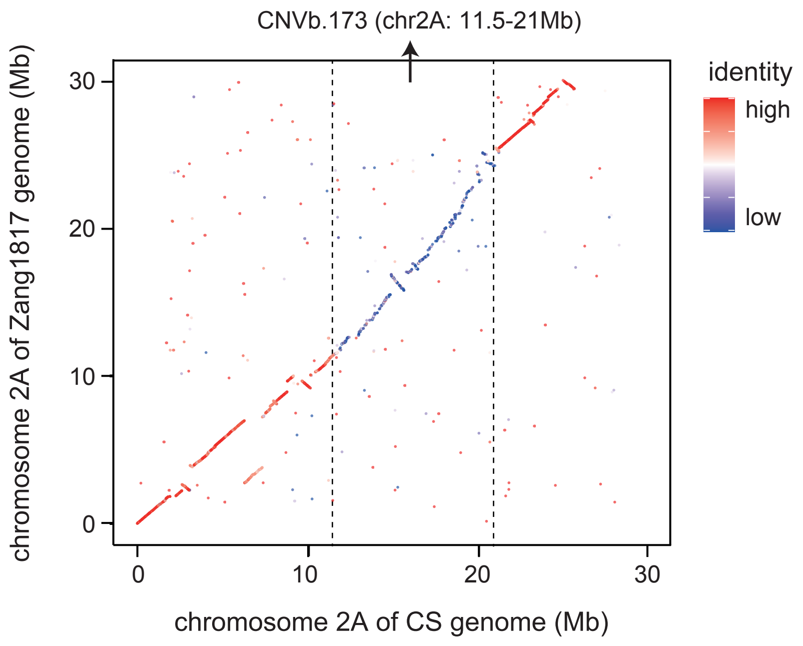

Fig S9

Comparative dot plot alignment of Zang1817 chromosome 2A and CS chromosome 2A (showing the first 30 Mb).

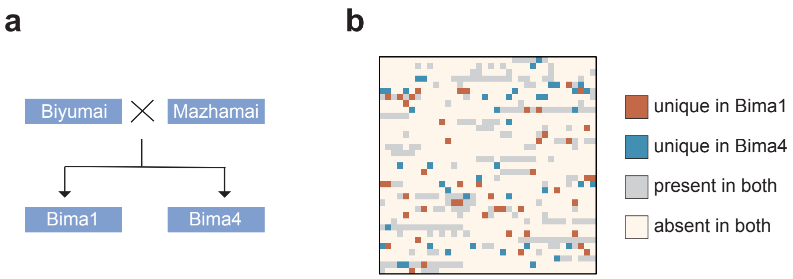

Fig S10

A paired comparison between Bima1 and Bima4. **a.** The pedigree diagram shows the breeding relationship between Bima1 and Bima4, originating from a cross between Biyumai and Mazhamai. **b.** The CNVb fingerprint of a paired co-mparison between Bima1 and Bima4. Each small square in the CNVb fingerprint denotes an individual CNVb marker.

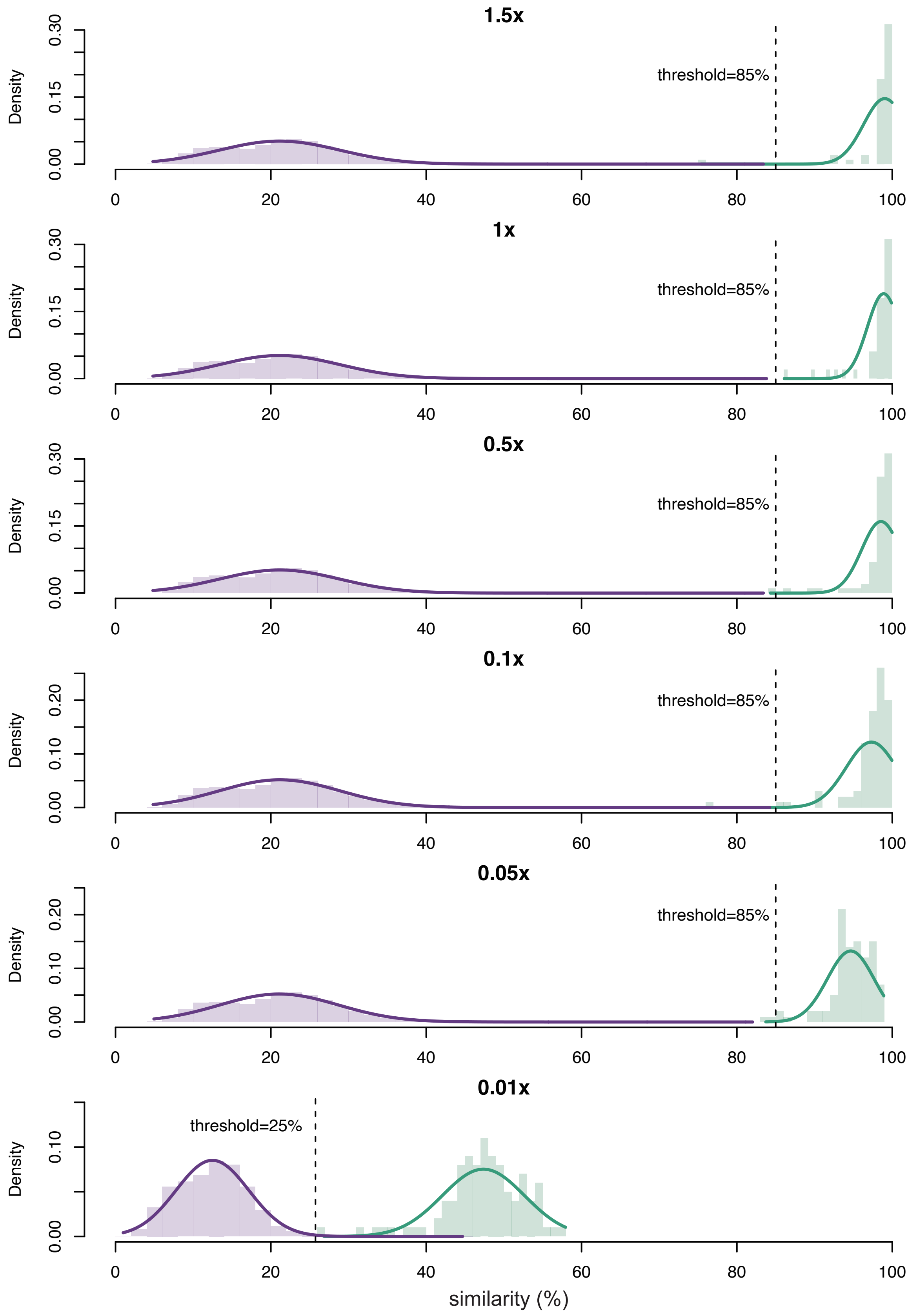

Fig S11

Pairwise similarity distribution based on CNVb markers at ultra-low sequencing depth. Green represents the similarity between the same variety, while purple represents the similarity between different varieties.

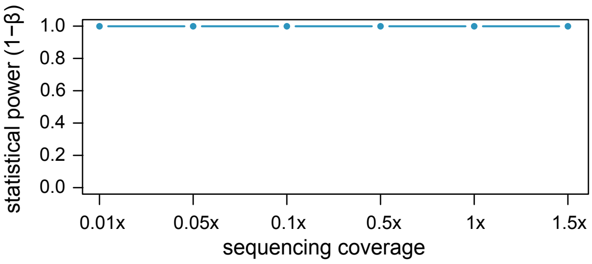

Fig S12

Statistical power (1-β) of the similarity density distributions for low-coverage sequencing datasets with different coverage.
