# Supplementary material for: Tagging large CNV blocks in wheat boosts digitalization of germplasm resources by ultra-low-coverage sequencing
Source: Genome Biol. 2024 Jul 1;25:171. doi: 10.1186/s13059-024-03315-6 (PMC11218387; doi:10.1186/s13059-024-03315-6)
Supplement: Supplementary file 3 — Additional file3 (DOCX 1516 KB) [file 13059_2024_3315_MOESM3_ESM.docx]

Review History

**First round of review**

**Reviewer 1**

**Were you able to assess all statistics in the manuscript, including the appropriateness of statistical tests used?**

Yes, and I have assessed the statistics in my report.

**Comments to author:**

In this manuscript, Niu and colleagues described a set of 1,240 large copy number variation blocks-based markers, allowing for accurate wheat germplasm identification even at ultra-low sequencing coverage. This set of markers was identified based on a panel of 528 worldwide wheat accessions with resequencing data available using a homemade pan-genome reference. Based on the marker set, the authors provided the CNVb genotypes across 1,171 accessions. Solid statistic comparison validated the method in the aspect of wheat germplasm identification. The authors also provide a well-designed website with multiple functions based on CNVb genotypes. As such, the manuscript provides a precise and cost-effective genotyping tool that could be highly utilized for the genetic characterization of wheat germplasm.
There are multiple points were unclear to me and need further clarification.
1. The authors used 528 wheat resequencing accessions to construct the marker set and provided the CNVb genotype across 1,171 global wheat accessions. It is unclear to me how the subset of wheat lines has been selected for the marker set construction, though the total count of accessions has been saturated. To show the representative of accessions included, it is suggested to label which accessions have been used for marker set construction in supplementary Table 1, and to include other passport information of germplasm, such as geographical locations.
2. "The construction of non-Chinese Spring chromosome" section and lacks details. How were the CNVb markers identification at pan-genome level, which is needed to be clarified. The "Development of CNVb markers" section is lengthy and not well structured. I suggest revising these two methods sections.
3. The WheatCNVb database provided in the manuscript offers a valuable feature that allows users to upload their interest accessions for customized CNVb analysis. Before uploading the data for further analysis, the genomic data should be pre-analyzed and processed to a specific data format. However, on the website, there lack of detailed guidelines or more comprehensive help documentation for this pre-analyzed step. Providing this guide will help non-experts in bioinformatics go through this step, and help this valuable resource be utilized by the wheat research community.
4. Line 237 mentioned that 1BL·1RS can be divided into two types, yet it is challenging to distinguish accessions with these two types from figure 2d.
5. In line 291, "By scanning the CNVb markers over the mapping read-depth profile, regardless of further SNPs/InDels calling, we can accurately generate the digitalized PAV map of CNVb markers". By using the word "regardless of", I think the authors intend to express "without the need to"?
6. In line 242, the length of pericentric introgression on chromosome 2B from Triticum timopheevii was stated as 427 Mb, yet line 245 indicated the corresponding CNVb marker interval as chr2B:89.5-769.0 Mb, which seems inconsistent in length. Please explain it.
4. The full spellings of some abbreviations are not provided across the manuscript, such as the term "PAV" mentioned in line 294. Please provide the full spelling of abbreviations at the first appearance in the manuscript.
8. It seems that the chromosome model of 4B, 1D, 1B and 2B are the same in Figure 3a, 3c, 3d and 3e. Please verify the relative positions of the centromeres across these 4 chromosomes.
9. Figure 5, "2NvS/2AS" should be formatted as"2NvS/2AS".

**Reviewer 2**

**Were you able to assess all statistics in the manuscript, including the appropriateness of statistical tests used?**

Yes, and I have assessed the statistics in my report.

**Comments to author:**

The manuscript provides a comprehensive investigation into the development and application of CNVb markers for precise genotyping and management of wheat germplasm resources. It underscores the benefits of CNV markers, emphasizing their accuracy and efficiency in identifying wheat varieties. The study reflects a significant commitment to detailed data analysis to overcome the complexities associated with the wheat genome. Despite these strengths, there are notable concerns regarding the broad applicability and certain technical aspects within the manuscript that warrant further discussion and clarification.
1. To comprehensively assess the broad applicability of CNVb markers in wheat genomics, it is essential to evaluate the current advancements in this field achieved through the development and utilization of CNVb markers. While the study focuses on comparing the recall rates between CNVb markers and SNP markers, as well as calculating the relatedness between CNVb and gIBD-based similarity, additional comparisons and discussions regarding CNVb markers in relation to current markers or technologies are necessary to fully elucidate their advantages in relevant field applications.
2. While the manuscript predominantly centers on the creation of copy number variation (CNV) markers, the analysis and discussion primarily revolve around present/absent variations, which represent just one specific type of CNV, overlooking other forms of copy number changes. Although the study did identify some CNVb markers as duplications, there is a lack of clarity on how these markers are defined in relation to read mapping coverage. It is crucial to elaborate on how the copy number of these markers is discriminated and how they are genotyped for subsequent application.
3. The rationale for assembling the pangenome and its significance in this study should be clearly explained, especially considering that most of the study does not directly relate to the pangenome. The authors claim that the pangenome can help mitigate the high rate of false positives and negatives in variant calling (line384); however, this assertion lacks specific evidence or validation that should be included. Additionally, what is the practical implications of identifying CNVb markers in non-CS segments.
4. The explanation of the pangenome assembly pipeline (lines 147-151) lacks clarity. The authors mention mapping resequencing data of Chinese Spring to other assembled references to generate novel genome blocks, suggesting the use of unmapped reads for assembly. However, the method section describes a different approach of mapping CS sequencing reads to other assemblies to identify blocks present in other assemblies but absent in CS. If the method involves mapping reads to identify deletion blocks, why not directly compare the whole genomes of CS and other assemblies to pinpoint the deletions unique to CS?
5. The authenticity of CNVb markers was confirmed through a limited number of PCR assays (line274), and the recall rate of CNVb markers with ultra-low-coverage sequencing was assessed against high-coverage sequencing data. However, with the availability of multiple de novo assembly references, I suggest conducting these validations using these references. This involves directly identifying the CNVb markers in the assembly to see if the markers is authentic or not, and also, utilizing ultra-low-sequencing data from these assembled varieties to evaluate how effectively these markers can be recalled.
6. The author noted the uneven distribution of CNV bins (Line 111). Observing Figure S6, it is evident that the distribution is not uniform. Do the authors have additional parameters to characterize the even distribution of CNVb markers? Furthermore, would these uneven distributions impact the downstream application of these markers?
7. What is the significance of linking CNVb markers with known beneficial alleles? Considering that CNVb covers a substantial portion of the genome, there are likely numerous associations between CNVs and specific alleles. However, many alleles are not directly related to CNV. For instance, in the case of the Glu-Db gene, the functional allele is not determined by the presence or absence of this gene. Therefore, I am curious about the biological rationale for associating them together in this context.
8, The authors discussed the identification of an inversion in chr6B (line211-213). I am curious about how the pipeline can detect inversions, as CNVb markers are established and genotyped based on the sequencing read coverage. However, inversions cannot be genotyped by examining the read coverage.
9. Line232 discusses the 1RS.1BL translocation. How can the pipeline reveal the translocation variation by analyzing and genotyping the CNVb marker?
10. Referring to Figure3f, Zang1817 exhibits a10 Mb deletion compared to CS. However, the collinearity results from Figure S8 indicate no deletion in the 11.5-21 Mb region, despite two small inversions within this interval. Additionally, in Line 273, the authors mentioned another marker (CNVb.142), and I am unsure about the relationship between CNVb.142 and CNVb.173.
11. In the method for identifying CNV blocks, the authors utilized the criterion of read counts below 0.5 or above 1.5 to distinguish between deletion and duplication windows. It is crucial to clarify whether a specific assay was employed to establish this criterion. Furthermore, it is important to investigate whether there are specific criteria in place for determining copy numbers exceeding 2 and how these high-copy number blocks will be accessed in the marker identification process and genotyped in subsequent applications. Additionally, Line 531 prompts questions about the criteria used for genotyping CNVb markers using low-coverage data. It is essential to delve into the details of this genotyping criterion and evaluate its effectiveness under the constraints of low-coverage conditions.

**Authors Response**

**Point-by-point responses to the reviewers’ comments:**

**Reviewer #1:**

In this manuscript, Niu and colleagues described a set of 1,240 large copy number variation blocks-based markers, allowing for accurate wheat germplasm identification even at ultra-low sequencing coverage. This set of markers was identified based on a panel of 528 worldwide wheat accessions with resequencing data available using a homemade pan-genome reference. Based on the marker set, the authors provided the CNVb genotypes across 1,171 accessions. Solid statistic comparison validated the method in the aspect of wheat germplasm identification. The authors also provide a well-designed website with multiple functions based on CNVb genotypes. As such, the manuscript provides a precise and cost-effective genotyping tool that could be highly utilized for the genetic characterization of wheat germplasm.

There are multiple points were unclear to me and need further clarification.

***Response:***

*We are immensely grateful for your positive and encouraging feedback on our manuscript.*

1. The authors used 528 wheat resequencing accessions to construct the marker set and provided the CNVb genotype across 1,171 global wheat accessions. It is unclear to me how the subset of wheat lines has been selected for the marker set construction, though the total count of accessions has been saturated. To show the representative of accessions included, it is suggested to label which accessions have been used for marker set construction in supplementary Table 1, and to include other passport information of germplasm, such as geographical locations.

***Response:***

*Thanks for your valuable suggestions. We have updated Supplementary Table 1 to clearly indicate which of the wheat accessions were utilized for the construction of the CNVb marker set. Additionally, we have offered comprehensive passport information on the germplasm, such as geographical location information for each accession, as recommended, to enhance the representativeness and utility of our dataset. The 528 accessions selected for the CNVb marker set construction, comprising 352 from China and 176 from other countries like the USA, Germany, and India, were chosen to ensure broad geographic diversity.*

2. “The construction of non-Chinese Spring chromosome” section and lacks details. How were the CNVb markers identification at pan-genome level, which is needed to be clarified. The “Development of CNVb markers” section is lengthy and not well structured. I suggest revising these two methods sections.

***Response:***

*Thank you again for the valuable suggestions. For the pan-genome construction, we've detailed the process of obtaining non-Chinese Spring sequence blocks, including the software and parameters used. we have made significant revisions to the “Construction of non-Chinese Spring chromosome” section as follows. “To construct the wheat pangenome, we first collected de novo assembled genomes of 17 wheat varieties [15, 26, 29-33], including the reference assembly of Chinese Spring RefSeq v1 (CSv1). Excluding CSv1, the remaining 16 genomes were ranked based on contig N50 values and whether Hi-C sequencing was used for assembly quality assessment (Additional file 1: Table S2). We identified sequences absent in the CS genome by aligning resequencing data from the 17 varieties against the 16 ranked reference assemblies. The alignment process involved trimming raw reads using Trimmomatic, followed by mapping high-quality reads to the wheat pan-genome with BWA-MEM [47]. Bamtools v2.4 [48] was used to filter read pairs with abnormal insert sizes (>10,000 bp or =0 bp) or low mapping quality scores (<1). Samtools v1.3 [49] was employed to remove potential PCR duplicate reads. Starting with the highest-ranked Aikang58 genome as* *the reference, we aligned CS resequencing data to Aikang58, using a 1 Mb sliding window and a specific method for identifying deletion blocks to detect sequences absent in CS relative to Aikang58. This procedure was iteratively applied, comparing CS and Aikang58 resequencing data against the second-ranked Fielder genome to identify non-redundant deletion blocks relative to Fielder, and continued through all 16 varieties. Through this methodology, we extracted non-redundant deletion block sequences absent in CS, which were assembled in chromosomal order into "chrNCP" as a supplementary genome sequence to the CS reference. Thus, "chrNCP" combined with the CS genome forms the wheat pan-genome* ***(Additional file 1: Table S4)****.”*

*In the development of CNVb markers, we restructured the section with clear subheadings (Step 1, Filtering of raw CNV blocks, Step 2, Merging CNV blocks, Step 3, Filtering CNVb markers for ulcWGS stability) to improve logical flow and clarity. The revision is as follows*.

*“To develop CNVb markers from 528 high-coverage resequenced datasets, the identification of raw CNV blocks was refined through a systematic process structured into three main steps.*

***Step 1: Filtering of raw CNV blocks*** *Initially, for CNV blocks aligned to the CS reference regions, we employed a multinomial Hidden Markov Model (HMM) using the hmmlearn Python library (https://pypi.org/project/hmmlearn/) to minimize random noise and enhance the clarity of CNV block patterns. This model was configured with parameters set to "n_components=3, n_iter=60, tol=0.001", and optimized via the Baum-Welch iterative re-estimation algorithm through the "fit()" method. The "decode()" method, with "algorithm=viterbi", was then used to smooth and decode CNV blocks. CNV blocks with a value of length / 100,000 + frequency ≤ 10 were further filtered out, where 'frequency' indicates the number of accessions containing the CNV block. For CNV blocks mapped to the "chrNCP" genome, a similar filtration and refinement were applied, excluding CNV blocks with a value of length / 1,000,000 + frequency ≤ 10 or length / 1,000,000 + 528 - frequency ≤ 10.*

***Step 2: Merging CNV blocks.*** *For CNV blocks within the CS reference regions,* *redundancy was addressed by merging significantly overlapping blocks (ρ_o_ ≥ 0.8) and merging linked blocks (those within 5 Mb apart and with ρ_link_ ≥ 0.9). The formulas for ρ_o_ and ρ_link_ are defined as:*

$\rho_{o}=\frac{L_{o}}{L_{1}+L_{2}-L_{o}}$

$\rho_{link}=\frac{C_{s}}{C_{1}+C_{2}-C_{s}}$

*where L_1_ and L_2_ are the lengths of the CNV blocks, L_o_ is the overlapping length, C_1_ and C_2_ are the counts of accessions carrying each CNV block, and C_s_ is the count of accessions with both CNV blocks. No further processing was needed for already filtered CNV blocks corresponding to the "chrNCP" genome. This merging step resulted in a preliminary CNV marker library, encompassing multiple CNV blocks per marker. Markers identified in both the CS reference and "chrNCP" sequences were assessed for redundancy with a specific focus on their presence or absence across accessions. If a marker identified in the CS region is consistently present across a subset of accessions while a corresponding marker identified in the "chrNCP" sequence is consistently absent in the same accessions, or vice versa, the marker from the "chrNCP" sequence will be filtered out.*

***Step 3: Filtering CNVb markers for ulcWGS stability.*** *To ensure the applicability of CNVb markers for ulcWGS data, markers indistinguishable at low sequencing coverage were excluded. CNV blocks were initially identified from hcWGS and simulated 0.1× coverage data, with the latter obtained by downsampling hcWGS data. Each accession’s CNV blocks were compared against the preliminary marker library to ascertain the presence or absence of CNVb markers in both hcWGS and 0.1× data. A marker was considered present if at least one CNV block overlapped by ≥ 90% and the length discrepancy was under 1 Mb. Markers with inconsistent detections in more than 10 accessions were removed. The refined set of CNVb markers formed the finalized marker collection.”*

*Additionally, we introduced a new subsection on “Identification of CNVb markers using ulcWGS” elaborating on the methodology and specific parameters for CNVb* *marker* identification as follows.

“The pipeline is to first identify the type of CNV blocks and then match these CNV blocks to the corresponding markers to identify which markers are present in each variety. Initially, the ulcWGS data are aligned to the pan-genome to detect raw deletion (copies=0) and duplication (copies≥2) blocks. These blocks are then mapped separately to their corresponding CNVb marker set—deletion blocks to the deletion marker set and duplication blocks to the duplication marker set. The presence of a deletion or duplication marker in a variety is determined based on the following criteria, if a deletion or duplication block present in the variety overlaps with a deletion or duplication marker by at least 90% and the difference in length between the block and the marker is less than 100 Kb, the deletion or duplication marker is considered present in that variety.”

3. The WheatCNVb database provided in the manuscript offers a valuable feature that allows users to upload their interest accessions for customized CNVb analysis. Before uploading the data for further analysis, the genomic data should be pre-analyzed and processed to a specific data format. However, on the website, there lack of detailed guidelines or more comprehensive help documentation for this pre-analyzed step. Providing this guide will help non-experts in bioinformatics go through this step, and help this valuable resource be utilized by the wheat research community.

***Response:***

*Thank you for highlighting the need for detailed guidelines and comprehensive help documentation for users uploading their genomic data to the WheatCNVb database.*

*In response, we have taken the following actions: (1) We have created detailed guidelines with step-by-step instructions and provided examples for the pre-analysis and data formatting process to ensure users can prepare their genomic data for upload successfully. (2) We have provided comprehensive help documentation, including tutorials, FAQs, and troubleshooting tips to assist users in navigating the WheatCNVb database. (3) We constructed a dedicated support section on WheatCNVb website, by* *making the guidelines and help documentation easily accessible. Users can access the guidelines, tutorials, and other support resources at* [*http://wheat.cau.edu.cn/WheatCNVb/tutorial.html*](http://wheat.cau.edu.cn/WheatCNVb/tutorial.html) ***(Figure R1)****. (4) We also provided contact information, for the potential technical support in the future. We anticipate these improvements will enhance accessibility and user experience, fostering greater use of the WheatCNVb database.*


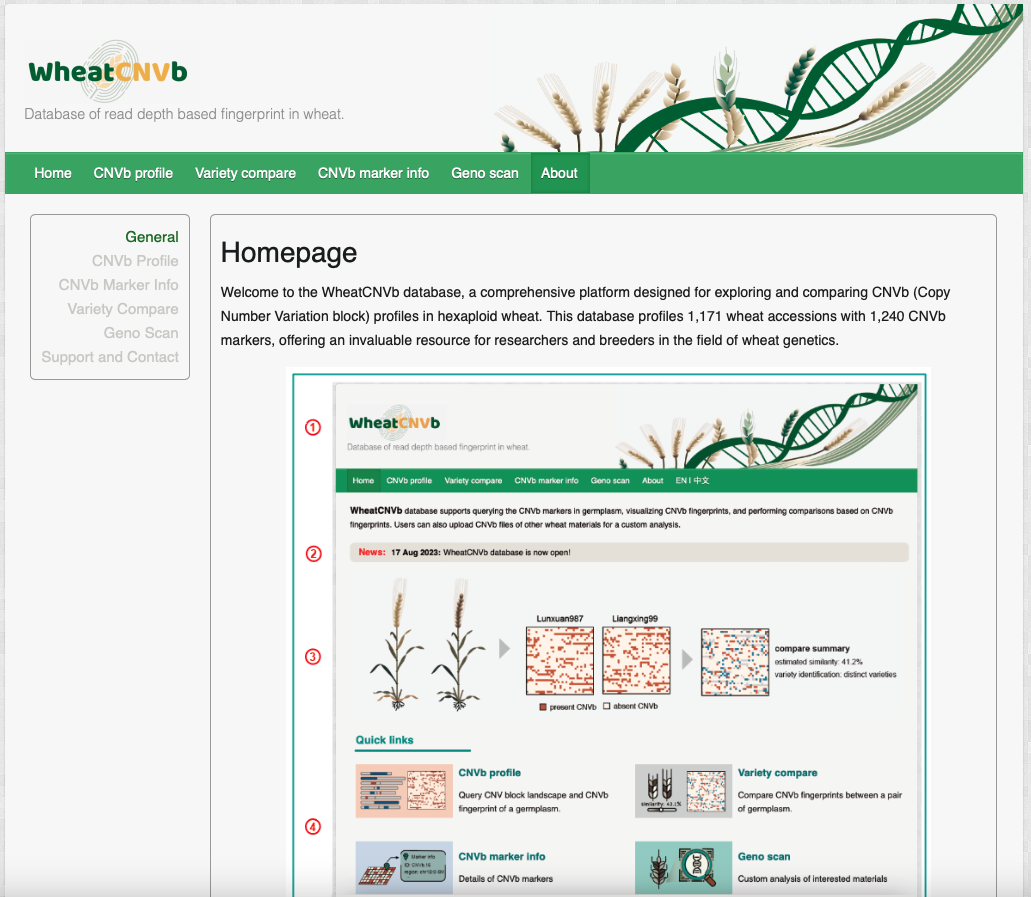


**Figure R1.** Screenshots of the tutorial webpage of WheatCNVb website.

4. Line 237 mentioned that 1BL·1RS can be divided into two types, yet it is challenging to distinguish accessions with these two types from figure 3d.

***Response:***

*Thanks for your feedback regarding the visibility of the differences between the two* *CNVb markers in* ***Figure 3d****.*

*We have addressed this concern by including a zoomed-in view of the markers in the* ***Figure S7*** *to clearly showcase the small interval difference. Additionally, in the main figure, we have now labeled the two markers as “type 1” and “type 2” to indicate their distinct types directly. This enhancement should facilitate easier distinction between the two types of 1BL·1RS accessions for readers.*


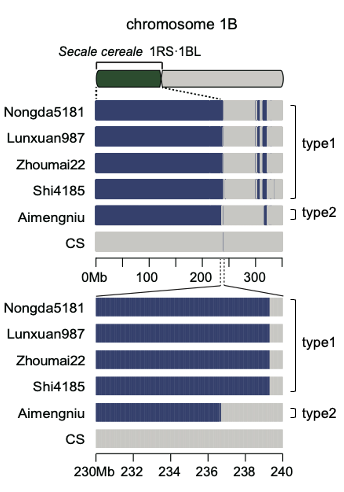


**Figure S7**. The CNVb-deletion marker associates with the 1RS·1BL translocation. The “type 1” denoted the subtype 1RS·1BL translocation associated with CNVb.67.1 (chr1B: 0-239.3 Mb). The “type 2” denoted the subtype 1RS∙7DL/7DS∙1BL translocation associated with CNVb.67.2 (chr1B:0-236.7 Mb).

5. In line 291, "By scanning the CNVb markers over the mapping read-depth profile, regardless of further SNPs/InDels calling, we can accurately generate the digitalized PAV map of CNVb markers". By using the word "regardless of", I think the authors intend to express "without the need to"?

***Response:***

*Thank you for pointing out the confusion caused by our wording in line 291.*

*We indeed intend to express the significance of "without the need to" to convey the idea that our method does not require further SNPs/InDels calling to generate the digitalized PAV map of CNVb markers. We have now revised this sentence to accurately reflect this intence by using “without the need to” instead of “regardless of” as follows, “By scanning the CNVb markers over the mapping read-depth profile, without the need to further SNPs/InDels calling, we can accurately generate the digitalized PAV map of CNVb markers.”*

6. In line 242, the length of pericentric introgression on chromosome 2B from *Triticum timopheevii* was stated as 427 Mb, yet line 245 indicated the corresponding CNVb marker interval as chr2B:89.5-769.0 Mb, which seems inconsistent in length. Please explain it.

***Response:***

*Thank you for pointing out the discrepancy. We appreciate the opportunity to clarify this matter for a better understanding of our findings.*

*The 427 Mb pericentric introgression refers to the segment from Triticum timopheevii in the LongReach Lancer genome [1], while the CNVb marker interval (chr2B:89.5-769.0 Mb, deletion) denotes the deletion length in LongReach Lancer relative to the reference genome. The observed discrepancy arises due to the 427 Mb introgression from Triticum timopheevii in the Lancer genome, which leads to a non-alignable region when Lancer is compared to reference Chinese Spring, thus presenting as a deletion over the interval of 89.5-769.0 Mb in the Chinese Spring 2B chromosome. The sequence absent in Lancer yet present in Chinese Spring does not correspond in length to the Triticum timopheevii introgression in LongReach Lancer, leading to the length discrepancies reported in the article.*

7. The full spellings of some abbreviations are not provided across the manuscript, such as the term "PAV" mentioned in line 294. Please provide the full spelling of abbreviations at the first appearance in the manuscript.

***Response:***

*Thanks for pointing out this mistake. We have provided the full spellings of all abbreviations, including “PAV”, at the first appearance. Additionally, we have converted rarely used acronyms to their full terms throughout the text to enhance readability and understanding.*

8. It seems that the chromosome model of 4B, 1D, 1B and 2B are the same in Figure 3a, 3c, 3d and 3e. Please verify the relative positions of the centromeres across these 4 chromosomes.

***Response:***

*Thank you for pointing out the issue of the chromosome models in* ***Figure 3****. We have corrected the relative positions of the centromeres for chromosomes 4B, 1D, 1B, and 2B, in the revised manuscript.*


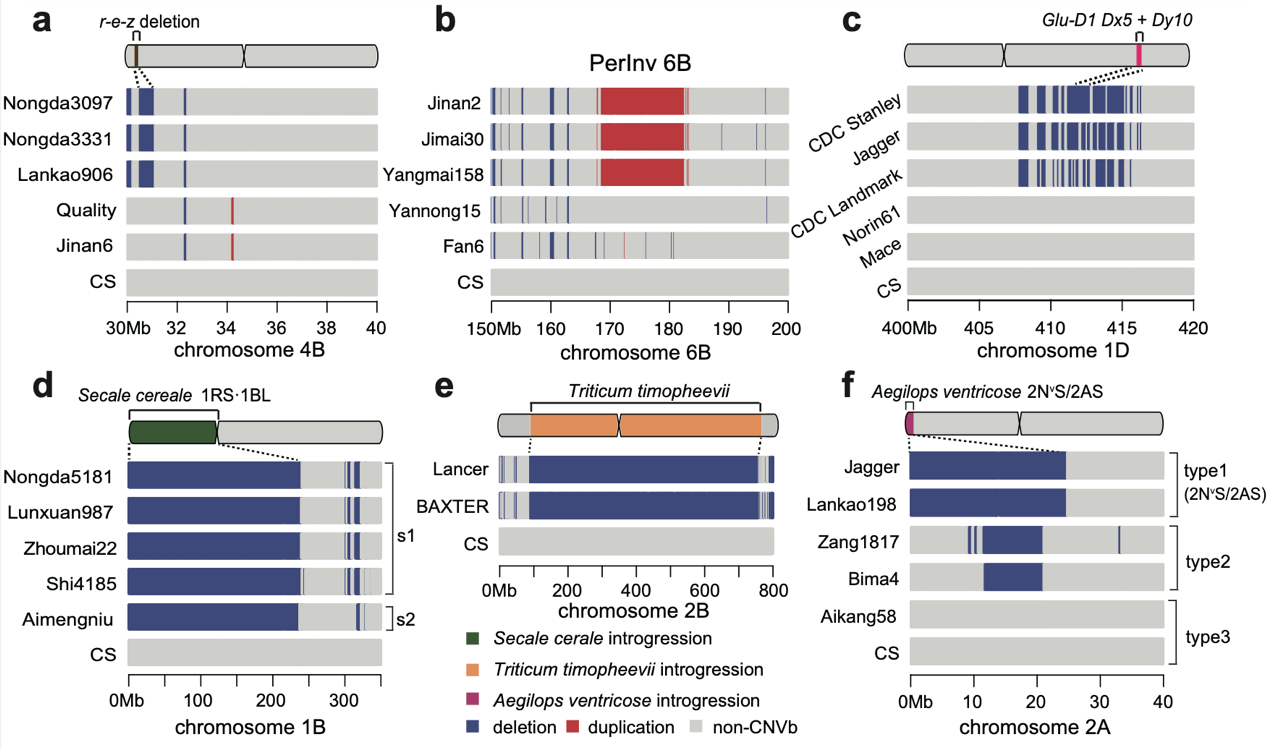


**Fig. 3** **Associating CNVb markers with known structural variations and predominant haplotypes.** **a.** The CNVb-deletion marker CNVb.647 (chr4B: 30.5-31.1 Mb) corresponds to the *r-e-z* haplotype on chromosome 4B. **b.** The CNVb.989 CNVb-duplication marker (chr6B: 167.9-183.4 Mb) is associated with the pericentric inversion on chromosome 6B (perInv 6B). **c.** The CNVb-deletion marker CNVb.162 (chr1D: 407.8-408.5 Mb) corresponds to the *Glu-D1d* (Dx5 + Dy10) allele of the high-molecular-weight glutenin gene *Glu-D1*. d. The CNVb-deletion marker CNVb.67.1 (chr1B: 0-239.3 Mb) associates with the 1RS·1BL translocation. **e.** The CNVb-deletion marker CNVb.290 (chr2B: 89.5-769 Mb) corresponds to the introgression from *Triticum timopheevii* on chromosome 2B. **f.** Distribution of three types of CNVb allelic genotypes within the first 40Mb region of chromosome 2A. The first type of CNVb allele (CNVb.189, chr2A: 0-24.7 Mb, deletion) corresponds to the introgressed segment from *Aegilops ventricose*.

9. Figure 5, "2NvS/2AS" should be formatted as"2N^v^S/2AS".

***Response:***

*Thank you for pointing out the formatting inconsistency in* ***Figure 5****. We have corrected "2NvS/2AS" to "2N^v^S/2AS" as suggested, ensuring the notation aligns with standard conventions.*

**Reviewer #2:**

The manuscript provides a comprehensive investigation into the development and application of CNVb markers for precise genotyping and management of wheat germplasm resources. It underscores the benefits of CNV markers, emphasizing their accuracy and efficiency in identifying wheat varieties. The study reflects a significant commitment to detailed data analysis to overcome the complexities associated with the wheat genome. Despite these strengths, there are notable concerns regarding the broad applicability and certain technical aspects within the manuscript that warrant further discussion and clarification.

***Response:***

*Thank you for your thorough review and valuable feedback on our manuscript, which have greatly aided us in refining the manuscript. We appreciate your recognition of the comprehensive investigation we conducted into the development and application of CNVb markers for the precise genotyping and management of wheat germplasm resources. We understand that you have raised concerns regarding the broad applicability of our findings and certain technical aspects of our study. We take these concerns seriously and have addressed each point in detail as follows.*

1. To comprehensively assess the broad applicability of CNVb markers in wheat genomics, it is essential to evaluate the current advancements in this field achieved through the development and utilization of CNVb markers. While the study focuses on comparing the recall rates between CNVb markers and SNP markers, as well as calculating the relatedness between CNVb and gIBD-based similarity, additional comparisons and discussions regarding CNVb markers in relation to current markers or technologies are necessary to fully elucidate their advantages in relevant field applications.

***Response:***

*Thank you for your valuable suggestions. According to your suggestion, we have compared CNVb with other technologies for germplasm discrimination in two aspects and revised discussions.*

*First, we compared the CNVb marker with KASP markers developed by Gao et al. [2] in the aspect of variety discrimination. Comparative analysis result showed that CNVb markers identified 150 differential markers between variety pairs, while KASP markers only identified an average of 40 differential markers for the same pairs of varieties (****Figure R2****), demonstrating a significantly higher resolution with CNVb markers in differentiating varieties.*


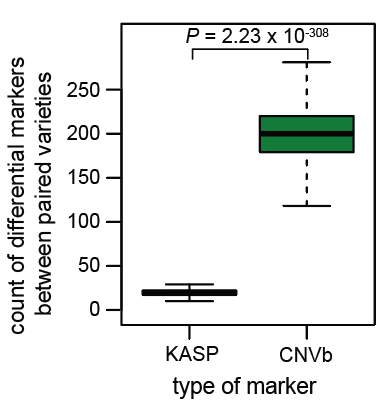


**Figure R2.** Comparison of the number of differential CNVb markers identified between wheat variety pairs using the KASP marker versus the CNVb marker.

*Second, we have comprehensively compared CNVb markers against four conventional types of markers (Southern blot-based, PCR-based, chip-based, and sequencing-based) in multiple aspects (****Table S9*** *in revised manuscript). We added a comprehensive comparison table of markers in* ***Table S9*** *in revised manuscript, and also revised the discussion of marker performance comparisons to be more comprehensive as follows.*

*“Compared to conventional methods for assessing wheat genetic resources in gene banks, CNVb markers showed multiple aspects of advantages* ***(Additional file 2: Table S9)****. First, CNVb markers significantly reduced the cost for genotyping per marker compared to Southern blot-based markers like RFLP and chip-based markers like SNP arrays, while being comparable in cost-effectiveness to SSRs and GBS. Second, CNVb markers support the utltra-low-depth high-throughput sequencing and can be fully automated, which is more labor-saving and less equipment-dependent than widely used SSR markers, making it more suitable for large-scale applications. Third, CNVb markers provide very high reliability, comparable to SNP arrays and better performance than GBS strategy. Fourth, CNVb markers provide high accuracy in variety identification, capable of distinguishing even closely related accessions (> 40%* *similarity), comparable to genome-wide gIBD analysis using high-coverage whole genome sequencing* ***(Fig. 4c)****. Fifth, CNVb markers support capturing larger genomic variations, which provides a unique layer of genetic information, and is crucial for identifying traits linked to structural variations. This feature is particularly advantageous in polyploid crops like wheat, where large genomic structural variations are prevalent. Thus, CNVb markers represent a low-cost, high-throughput, labor-saving, and highly reliable tool for modern breeding and germplasm management.”*

**Table S9**. Comparative overview of major molecular marker classes.

|  | RFLP | AFLP | SSR | SNP array | GBS | DArT | **CNVb** |
| --- | --- | --- | --- | --- | --- | --- | --- |
| Running cost | high | high | low | high | medium | high | **low** |
| Throughput | low | low | medium | high | very high | very high | **very high** |
| Automation level | low | low | medium | high | high | high | **high** |
| Reliability | high | medium | high | very high | high | high | **very high** |

RFLP, Restriction Fragment Length Polymorphism. AFLP, Amplified Fragment Length Polymorphism. SSR, Simple Sequence Repeat. SNP, Single Nucleotide Polymorphism. GBS: Genotyping By Sequencing. DArT, Diversity Arrays Technology. CNVb: Copy Number Variation Block.

2. While the manuscript predominantly centers on the creation of copy number variation (CNV) markers, the analysis and discussion primarily revolve around present/absent variations, which represent just one specific type of CNV, overlooking other forms of copy number changes. Although the study did identify some CNVb markers as duplications, there is a lack of clarity on how these markers are defined in relation to read mapping coverage. It is crucial to elaborate on how the copy number of these markers is discriminated and how they are genotyped for subsequent application.

***Response:***

*Thanks for pointing this out. To make the related content clearer, we have provide our* *responses and revised the manuscript as follows.*

*Firstly, the CNVb markers in our study can actually represent multiple forms of CNV status, and were classified into two main groups, that are, duplication (≥2 copies) and deletion (0 copies) CNVb markers. In previous manuscript, we defined the present/absent variations of CNVb markers by whether an accession harbors a specific CNVb marker or not, which is different from the PAV of sequences. To avoid misunderstanding, we revised the manuscript as follows.*

*“By scanning the CNVb markers over the mapping read-depth profile, without the need to further SNPs/InDels calling, we can accurately generate the digitalized map for presence and absence of CNVb markers (including deletion and duplication markers). When a CNVb marker is a duplication marker, its presence in a variety indicates that the variety contains this duplication block. Conversely, if a CNVb marker is a deletion marker, its presence indicates that the variety contains this deletion block.”*

*Secondly, we effectively utilize different types of CNVs in variety identification. it is important to note that together with normal marker (neither duplication nor deletion), we are possible to identify multiple alleles of CNVb markers with different copy numbers for one region. For instance, in the 0-25Mb region of chromosome 2B, Pingyang27 is identified with a deletion marker CNVb.295 (chr2B:276.9-290.8Mb, 0 copies). In the same region, Jimai19 carries duplication marker CNVb.336 (chr2B:276.9-290.8Mb, ≥2 copies). Lovrin10, showing neither CNVb.295 nor CNVb.336, is identified as having a normal copy number (1 copy). Thus, the presence of different CNV types in this region contributes to the resolution in distinguishing these accessions as different varieties during variety identification.*

*To provide clarity on our genotyping process for CNVb markers, a new subsection titled “Identification of CNVb markers using ulcWGS” has been added to the Methods* ***(Additional file 2: Figure S4)****. This subsection details the pipeline as follows.*

*“The pipeline is to first identify the type of CNV blocks and then match these CNV* *blocks to the corresponding marker set to identify which markers are present in each variety. Initially, the ulcWGS data are aligned to the pan-genome to detect raw deletion and duplication blocks. These blocks are then mapped separately to their corresponding CNVb marker set—deletion blocks to the deletion marker set and duplication blocks to the duplication marker set. The presence of a deletion or duplication marker in a variety is determined based on the following criteria, if a deletion or duplication block present in the variety overlaps with a deletion or duplication marker by at least 90% and the difference in length between the block and the marker is less than 100 Kb, the deletion or duplication marker is considered present in that variety.”*


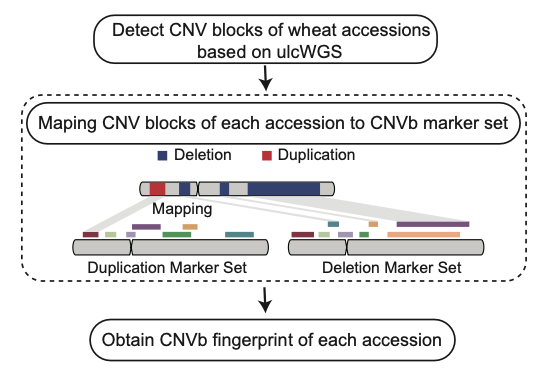


**Figure S4.** The Workflow for CNVb marker scanning in wheat varieties based on ulcWGS. The process begins with the detection of CNV blocks in wheat accessions utilizing ulcWGS. Subsequently, identified CNV blocks of each accession are mapped against the CNVb marker library. If a CNVb shows significant overlap with a CNVb marker, the wheat accession is considered to carry that specific CNVb marker. The collective mapping of these CNV blocks to the CNVb markers enables the establishment of a CNVb fingerprint for each accession, which is a composite representation of the presence or absence of specific CNVb markers in the genome.

3. The rationale for assembling the pangenome and its significance in this study should be clearly explained, especially considering that most of the study does not directly relate to the pangenome. The authors claim that the pangenome can help mitigate the high rate of false positives and negatives in variant calling (line 384); however, this assertion lacks specific evidence or validation that should be included. Additionally, what are the practical implications of identifying CNVb markers in non-CS segments.

***Response:***

*Thanks for your valuable suggestions. The significance of assembling a pangenome in this study is to minimize the biases associated with using Chinese Spring as the sole reference genome and to acquire a sufficient number of genetic markers. Previous studies have highlighted the extensive chromosomal rearrangements and frequent introgressions within bread wheat [3, 4, 5], indicating substantial genetic polymorphism. Consequently, single reference genome assemblies, such as Chinese Spring (CS), would not be able to encompass the full spectrum of genomic diversity within the species [6]. Our constructed pan-genome has incorporated approximately 2.6 Gb of non-CS genomic sequences, successfully capturing a significant amount of genetic polymorphisms absent in the CS reference genome. We have revised the manuscript to more clearly clarify the rationale and significance of constructing the pan-genome as follows.*

*“To mitigate the potential bias of CNVb identification introduced by mapping against a single reference genome and to acquire a sufficient number of genetic markers, we constructed a pan-reference genome by iterative mapping of the whole-genome resequencing data against 16 de novo assembled reference genome sequences [15, 26, 29-33].”*

*The pangenome is vital for this study, and the practical significance of identifying CNVb markers in non-CS segments is twofold: (1) CNVb markers in non-CS segments improved resolution of variety identification. By comparing CNVb markers identified using the CS genome versus those identified using the pan-genome. A random selection of 100 wheat variety pairs showed an average of 136 differential CNVb markers when using CS as a reference, compared to 197 differential CNVb markers when using the pan-genome (****Figure R3****). This confirms that using the pan-genome as a reference captures more CNVb markers and enhances the resolution of variety identification. (2)* *CNV blocks from non-CS segments covered genomic regions residing key genes significantly influence traits such as disease resistance. For instance, a segment from Thinopyrum ponticum on chromosome 3D of the LongReach Lancer genome (chrNCP-chr3D: 75-128Mb) carries important resistance genes Lr24 and Sr24 for leaf and stem rust [1], respectively. Additionally, a 34Mb introgression from Aegilops ventricosa on chromosome 2A of the Renan genome (chrNCP-chr2A:74-108Mb) includes multiple resistance genes (Yr17/ Lr37/ Sr38/Cre5) [7]. Thus, the construction of the pan-genome not only captures additional genetic diversity but also improves the accuracy of variety identification. We have revised the manuscript to more clearly clarify the practical significance of constructing the pan-genome as follows.*

*“We generated a comprehensive reference catalog of CNV blocks at the pan-genome level, which captures sequence polymorphisms absent in Chinese Spring and provides sufficient CNVb markers to perform accurate variety identification.”*

*Thank you for pointing out the confusion caused by the sentence in line 384 of the manuscript, which may have implied a causal relationship between the generation of a pan-genome and addressing higher rates of false positives and negatives in variant calling for ulcWGS. Actually, false positives and negatives associated with CNV block calling in ulcWGS are addressed by refining and merging raw CNV blocks. We have revised the sentence in the manuscript as follows.*

*“We generated a comprehensive reference catalog of CNV blocks at the pan-genome level, which captures sequence polymorphisms absent in Chinese Spring and provides sufficient CNVb markers to perform accurate variety identification. Additionally, we concurrently addressed the higher rates of false positives and negatives in CNV block calling specific to ulcWGS by refining and merging raw CNV blocks.”*


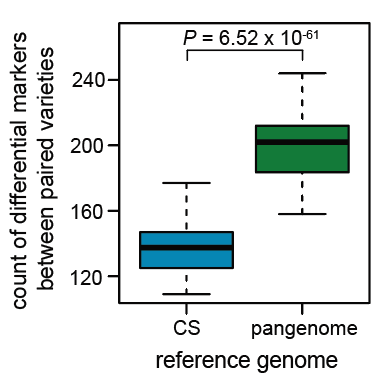


**Figure R3**. Comparison of the number of differential CNVb markers identified between 100 randomly selected wheat variety pairs using the CS reference genome versus the pan-genome.

4. The explanation of the pangenome assembly pipeline (lines 147-151) lacks clarity. The authors mention mapping resequencing data of Chinese Spring to other assembled references to generate novel genome blocks, suggesting the use of unmapped reads for assembly. However, the method section describes a different approach of mapping CS sequencing reads to other assemblies to identify blocks present in other assemblies but absent in CS. If the method involves mapping reads to identify deletion blocks, why not directly compare the whole genomes of CS and other assemblies to pinpoint the deletions unique to CS?

***Response:***

*Thanks for pointing out the unclear explanation. Following the reviewer’s suggestion, we have revised the explanation of the pan-genome assembly pipeline (lines 147-151) for clarity as follows.*

*“To mitigate the potential bias of CNVb identification introduced by mapping against a single reference genome and to acquire a sufficient number of genetic markers, we constructed a pan-reference genome by iterative mapping of the whole-genome* *resequencing data against 16 de novo assembled reference genome sequences [15, 26, 29-33]. This iterative process involved using a 1 Mb sliding window to precisely identify sequences that are present in these other genomes but absent in CS. Starting with the highest-ranked Aikang58 genome as the initial reference, and progressing through each of the genomes in order of their assembly quality, we systematically detected and compiled 975 novel genome blocks with a total length of 2.6 Gb* ***(Additional file 1: Table S3)****. These blocks, which represent genomic regions absent in CS, were then ordered chromosomally and assembled into the supplementary genome sequence denoted as 'chrNCP'* ***(Fig. 2a)****.”*

*The rationale for not directly comparing the CS genome with other assemblies to construct the pan-genome is threefold: (1) Genome alignment will identify significantly divergent segments as collinear regions that could not be mapped by short reads. Large introgressed or translocated segments that are in other varieties when compared to CS can be aligned to homologous regions in CS using whole genome alignment, resulting in the fragmentation of complete sequences. In contrast, aligning resequenced CS data to other genomes highlights large blocks that fail to align due to substantial divergence, thus accurately identifying these complete sequences as absent in CS. (2) Genomic block-based detection could identify larger CNV blocks, which are detected more reliably than smaller ones at low depths (****Figure R4a****). Whole-genome comparison to identify CS deletions, such as between the Zang1817 and CS assemblies, shows that 99.5% of the deletion intervals are shorter than 20Kb (****Figure R4b****). The approach of genomic block-based detection aligns with our ultimate goal of accurately identifying CNVb markers using ulcWGS. (3) The method of iteratively aligning resequenced data against assembled genomes is more cost-efficient. It also reduces the time cost compared to whole-genome comparisons. These points ensure our approach both captures significant genomic variations specific to CS and is suited to our intended downstream applications.*


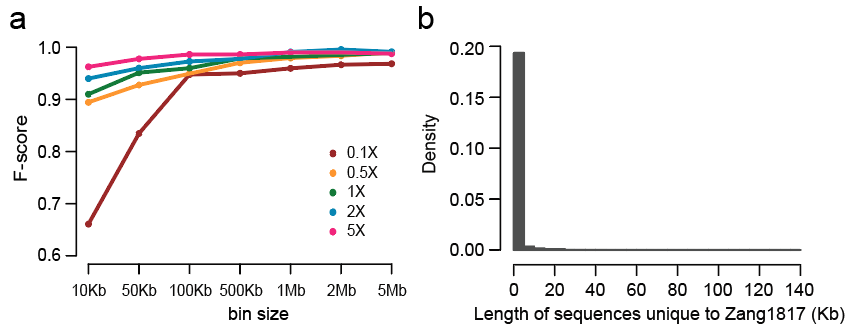


**Figure R4**. **a.** the F-scores for CNV bin identification at varying levels of sequencing depth (0.1×, 0.5×, 1×, 2×, 5×) using different window sizes (10Kb, 50Kb, 100Kb, 500Kb, 1Mb, 2Mb, 5Mb). **b.** Density distribution histogram of sequence lengths unique to Zang1817 compared to the CS reference genome.

5. The authenticity of CNVb markers was confirmed through a limited number of PCR assays (line 274), and the recall rate of CNVb markers with ultra-low-coverage sequencing was assessed against high-coverage sequencing data. However, with the availability of multiple de novo assembly references, I suggest conducting these validations using these references. This involves directly identifying the CNVb markers in the assembly to see if the markers is authentic or not, and also, utilizing ultra-low-sequencing data from these assembled varieties to evaluate how effectively these markers can be recalled.

***Response:***

*Thank you for your valuable suggestions. As a response, we conducted comparative genomic analysis using de novo assembly data from Mace and Jagger genomes to validate the accuracy of our CNVb marker identification. Three CNVb markers were verified as the example: a deletion marker on chromosome 1B from the Mace genome (CNVb.54, chr1B:123.9M-129.5M, deletion), a deletion marker on chromosome 1B from the Mace genome (CNVb.107, chr1B:314.6M-322.4M, deletion), and a deletion marker on chromosome 3D from the Jagger genome (CNVb.530, chr1B:570.6M-571.9M, deletion)* ***(Figure R5)****. All the three CNVb markers could be identified in the comparative assemblies, confirming their presence and the reliability of CNVb markers derived from read-depth analysis.*

*Furthermore, when ultra-low-coverage sequencing data (~0.05×) from Mace and Jagger were aligned to the CS reference genome, these CNVb markers were all successfully recalled, corroborating the accuracy of our CNVb marker identification process even at low sequencing depths.*


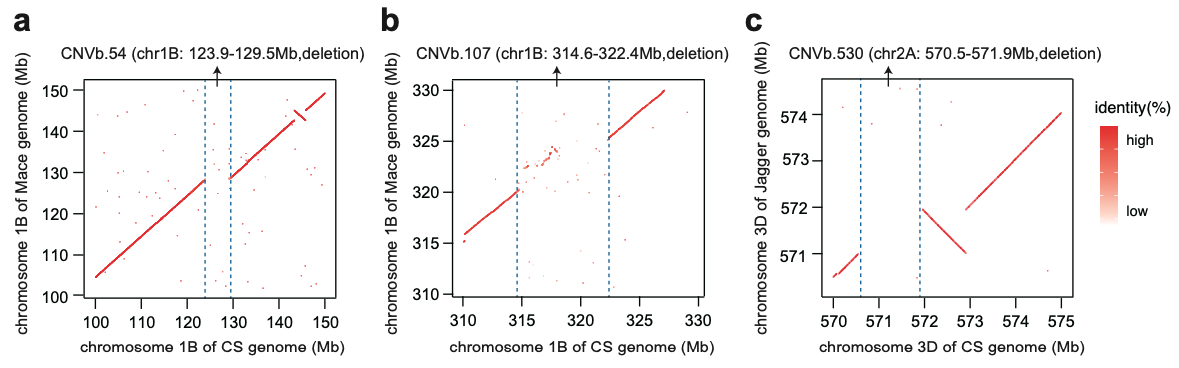


**Figure R5.** Comparative dot plot alignment of genome assembly and CS assembly. a. Comparative dot plot alignment between Mace chromosome 1B and CS chromosome 1B (100-150 Mb was shown). b. Comparative dot plot alignment of Mace chromosome 1B and CS chromosome 1B (showing the 310-330 Mb). c. Comparative dot plot alignment between Jagger chromosome 3D and CS chromosome 3D (570-575 Mb was shown). The blue dashed lines mark the boundary of the CNVb marker.

6. The author noted the uneven distribution of CNV bins (Line 111). Observing Figure S6, it is evident that the distribution is not uniform. Do the authors have additional parameters to characterize the even distribution of CNVb markers? Furthermore, would these uneven distributions impact the downstream application of these markers?

***Response:***

*Thanks for the comment. Following the reviewer’s concern, We further characterized the distribution of CNVb markers. The density of CNVb markers across the genome is sufficient for robust genotyping applications, although their distribution is not entirely* *uniform. The CNVb markers are abundant, with each chromosome featuring a range of 21~126 markers (averaging 59) per chromosome, thus will be able to provide comprehensive genomic insights. Moreover, our saturation analysis of CNVb markers suggests that the current maker set is nearing saturation* ***(Fig. 2c)****, further attesting to their utility for genomic characterization and variety identification.*

*Our analysis has shown that the uneven distribution of CNVb markers has no adverse impact on the downstream applications. For example, CNVb fingerprinting based on low-coverage sequencing can accurately differentiate and identify wheat varieties, both in selecting varieties for CNVb marker screening and in varieties not involved in the construction of the CNVb marker set* ***(Fig. 4)****.*


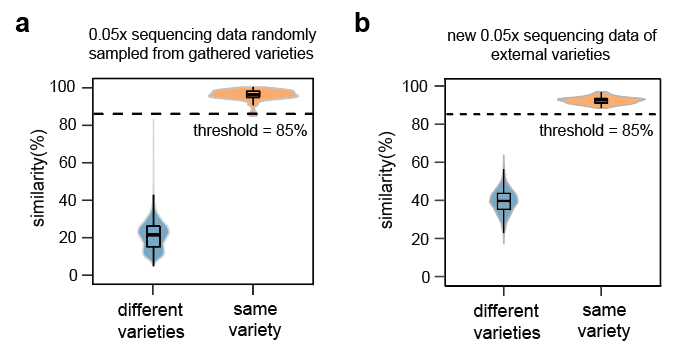


**Figure 4**. **a.** Similarity of pairwise accessions from two batches of 0.05× simulated sequencing datasets, each comprising 100 randomly selected accessions from our dataset. The dashed line represents the threshold (85%) for variety identification. **b.** The similarity of pairwise accessions from two batches of 0.05× sequencing data, each containing 100 accessions not included in the original CNVb marker library. The dashed line represents the similarity threshold (85%) for variety identification.

7. What is the significance of linking CNVb markers with known beneficial alleles? Considering that CNVb covers a substantial portion of the genome, there are likely numerous associations between CNVs and specific alleles. However, many alleles are not directly related to CNV. For instance, in the case of the Glu-Db gene, the functional allele is not determined by the presence or absence of this gene. Therefore, I am curious about the biological rationale for associating them together in this context.

***Response:***

*Thanks for your valuable commit. The significance of associating CNVb markers with known beneficial alleles includes two points: (1) Associating CNVb markers with known beneficial alleles enhances our understanding of breeding varieties that carry these alleles. “Introgression breeding” utilizing wild relatives for wide hybridization to introduce genetic diversity, particularly for traits like disease resistance, is an essential strategy in wheat breeding programs [3]. Many known beneficial alleles used in wheat breeding are derived from large structural variations, such as the 1BL·1RS and 2N^v^S translocations. (2) Associating CNVb markers with known beneficial alleles facilitates their utilization in breeding programs. These beneficial alleles or haplotypes are usually identified using complex techniques such as FISH or require high-quality genome sequencing, making their detection challenging in large populations at low cost. Thus, we associate beneficial alleles with CNVb markers to provide a high-throughput and cost-effective strategy for detecting and utilizing these alleles that significantly enhance desirable traits in wheat. According to your suggestion, we have revised and supplemented the manuscript to better explain the significance of associating beneficial alleles with CNVb markers as follows.*

*“To fully harness the potential of wheat carrying beneficial alleles such as disease resistance alleles for breeding applications, we have linked numerous well-known structural variations and beneficial alleles with CNVb markers.”*

*The logic for linking CNVb markers with beneficial alleles primarily consists of two* *points: (1) The high differentiation between beneficial alleles and reference segments leads to reduced read depth after alignment, thereby identifying the region as a deletion block. (2) There is a significant correlation between CNVb markers and beneficial alleles in varieties that have been previously identified by studies as carriers of these* *alleles. We have revised and supplemented the manuscript to better explain the biological rationale for associating beneficial alleles with CNVb markers as follows.*

*“We have linked numerous well-known structural variations and beneficial alleles with CNVb markers based on the high differentiation of beneficial alleles and reference sequences, as well as the significant correlation between CNVb markers and beneficial alleles.”*

*The logic for associating CNVb markers with the Glu-D1d (Dx5 + Dy10) haplotypes is twofold: (1) The Glu-D1d (Dx5 + Dy10) haplotype exhibits significant sequence divergence compared to the corresponding sequence in the CS genome. Previous study revealed that different haplotypes of Glu-D1d in bread wheat, primarily the Glu-D1a (Dx2 + Dy12) and Glu-D1d (Dx5 + Dy10) haplotypes, are highly differentiated [8]. In the CS reference genome, which carries the Glu-D1a (Dx2 + Dy12) haplotype, varieties with the Glu-D1d (Dx5 + Dy10) haplotype show significant sequence divergence at this sequence, making alignment difficult and appearing as reduced read depth, thereby identifying as a deletion block. (2) There is a significant correlation between CNVb markers and Glu-D1d (Dx5 + Dy10) haplotypes. We have detected a consistent deletion block marker among varieties carrying the Glu-D1d (Dx5 + Dy10) haplotype* ***(Figure R6)****. Comparing our CNVb marker-based identification with previously identified varieties carrying this haplotype, we found a full correlation. Thus, varieties with this deletion block marker are considered to carry the Glu-D1d (Dx5 + Dy10) haplotype.*


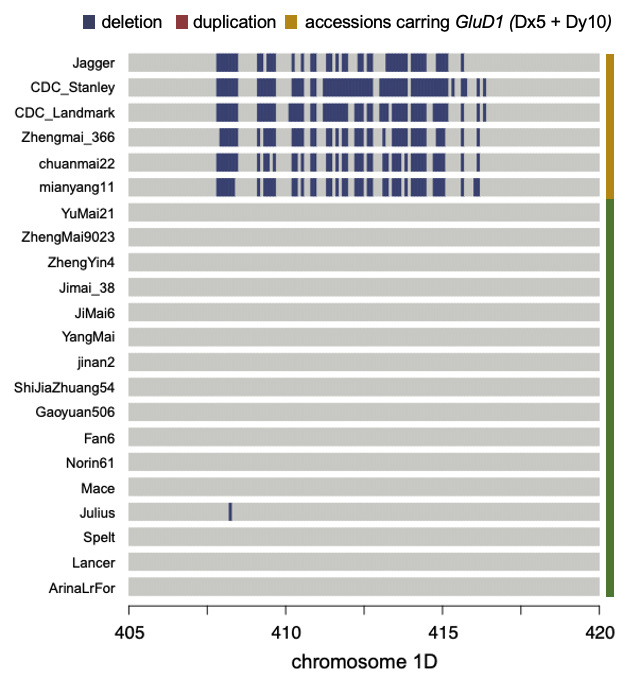


**Figure R6.** Visualization of CNVb distribution along chromosome 1D (405Mb-420Mb) across wheat varieties. Each row represents a variety, with yellow rectangles on the right indicating varieties carrying the *Glu-D1d* (Dx5 + Dy10).

8. The authors discussed the identification of an inversion in chr6B (line 211-213). I am curious about how the pipeline can detect inversions, as CNVb markers are established and genotyped based on the sequencing read coverage. However, inversions cannot be genotyped by examining the read coverage.

***Response:***

*Thanks for your valuable comments. Following the reviewers’ concern, we characterized a CNVb marker associated with the 6B inversion by identifying a shared duplication block marker that is significantly correlated with varieties carrying the 6B inversion, rather than by directly detecting the 6B inversion by examining the read coverage. We identified a shared duplication block marker in varieties known to contain* *the inversion on chromosome 6B* ***(Figure R7)****. Huang et al. identified certain varieties with the 6B inversion and others without it using Fluorescence in situ hybridization [9]. By comparing our study with Huang's findings among the same set of varieties, we found a consistent duplication block marker present in all varieties with the 6B inversion but absent in those without it. Therefore, we infer a full association between this duplication block marker and the 6B inversion. Thus, varieties with this CNVb marker are considered to carry the 6B inversion.*

*We have revised and supplemented the manuscript to better explain the significance and the biological rationale for associating the perInv 6B with CNVb markers as follows.*

*“We successfully associated a CNVb-duplication marker (CNVb.989, chr6B:167.9-183.4 Mb) on chromosome 6B with the perInv 6B based on our identification of a shared duplication block marker that is significantly correlated with varieties carrying the 6B inversion* ***(Fig. 3b).****”*


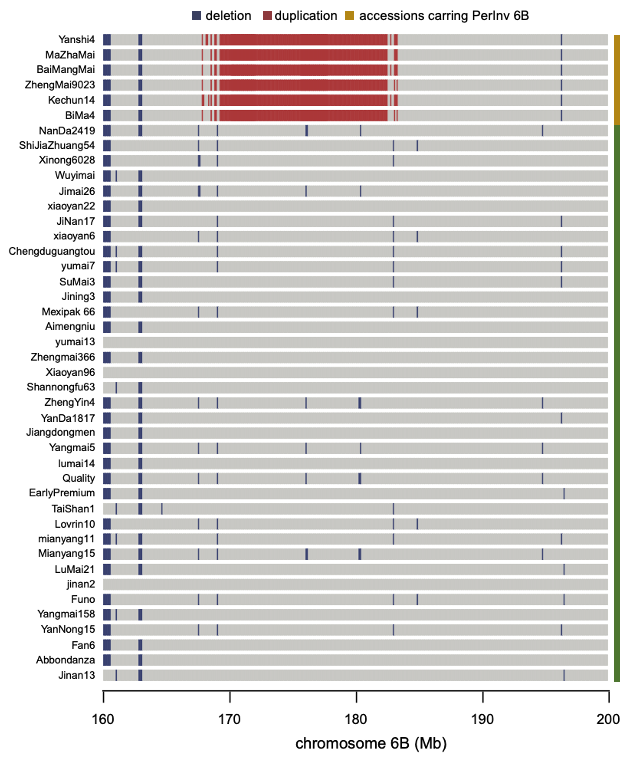


**Figure R7.** Visualization of CNVb distribution along chromosome 6B (160Mb-200Mb) across wheat varieties. Each row represents a variety, with yellow rectangles on the right indicating varieties identified by Huang et al. as carrying the 6B inversion.

9. Line232 discusses the 1RS·1BL translocation. How can the pipeline reveal the translocation variation by analyzing and genotyping the CNVb marker?

***Response:***

*Thanks for your helpful comments. The biological rationale for associating CNVb* *markers with the 1RS·1BL translocation is twofold as follows.*

*First, there is a high divergence of the translocated segment from the corresponding sequence in the CS reference genome. The 1RS·1BL translocation involves a high divergent segment from the short arm of the rye chromosome 1R translocated to the short arm of wheat chromosome 1B. When aligned to the CS reference genome, the 1RS segment shows reduced sequence homology, resulting in poor alignment and, consequently, lower sequencing coverage, which is then interpreted as a deletion* ***(Figure R8).*** *This finding is supported by Yang et al. [5], who identified CNV-deletions across the entire 1BS chromosome in wheat varieties containing the 1RS·1BL translocation. Thus, we can leverage the CNVb-deletion marker to identify high divergent translocated segment.*

*Second, there is a significant correlation between CNVb markers and 1RS·1BL translocation. (1) There is a significant correlation between CNVb markers and 1RS translocated segment on chrNCP. During the construction of the pan-genome, the 1RS translocated segment from Aikang58, absent in CS, was extracted to build the pan-genome. It was observed that varieties possessing the 1BS deletion block consistently identified the corresponding 1RS translocated block on chrNCP when aligned, underscoring the accuracy of using this 1BS deletion marker to detect the 1RS·1BL translocation. (2) The varieties identified as carrying the 1RS·1BL translocation using our CNVb marker approach are consistent with those identified by Yang et al. [5], through sequencing, and Huang et al. [9], via Fluorescence in situ hybridization.*

*We have revised and supplemented the manuscript to better explain the biological rationale for associating the 1RS·1BL translocation with CNVb markers as follows.*

*“In this study, we further developed CNVb-deletion markers (CNVb.67.1, chr1B: 0.1-239.3 Mb, deletion) for representing the 1RS utilizations based on the high divergence of the translocated segment from the corresponding sequence in the CS reference genome* ***(Fig. 3d)****. The CNVb.67.1 was significant correlated with 16 accessions that were convinced as harboring the 1RS·1BL translocation by fluorescence in situ hybridization [20], including Lunxuan987.”*


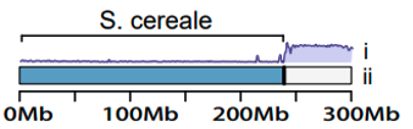


**Figure R8**. CNVb distribution on the first 300Mb of chromosome 1B in Aikang58. The graph depicts read depth (i) and the locations of CNV blocks (ii) along the chromosome.

10. Referring to Figure 3f, Zang1817 exhibits a 10 Mb deletion compared to CS. However, the collinearity results from Figure S8 indicate no deletion in the 11.5-21 Mb region, despite two small inversions within this interval. Additionally, in Line 273, the authors mentioned another marker (CNVb.142), and I am unsure about the relationship between CNVb.142 and CNVb.173.

***Response:***

*Thank you for pointing out the confusion caused by the statement, and we have added a table as well as updated the related figure* ***(Figure S8 and Table R2)*** *to accurately represent differences between Zang1817 and CS. This 10 Mb segment is a high differentiation level (only with 93% similarity) segment, resulting in the identification of a deletion in the 11.5-21 Mb region when aligning Zang1817 to the Chinese Spring reference genome. This deletion was not readily apparent in previous collinearity results. To address this, we have modified the dot plot of collinearity between Zang1817 and the Chinese Spring 2A chromosome. The revised plot shows low identity in the 11.5-21 Mb region* ***(Figure S8)****, confirming the presence of a translocated segment in Zang1817 and its identification as a deletion upon alignment with CS. We conducted a Nucleotide BLAST search to identify potential species sources for the translocated segment located on chromosome 2A (11.5-21 Mb) of the Zang1817 genome, which suggests that the segment possibly originates from Triticum monococcum* ***(Table R2)****. Thus, the identification of the deletion in the 11.5-21 Mb region is due to the high differentiation level of a possible translocation from Triticum monococcum and poor alignment with the reference genome sequence.*

*Furthermore, we apologize for any confusion caused by the mention of another marker* *(CNVb.142) in line 273. This was an error in our text and the correct marker associated with the described deletion is CNVb.173. We have corrected "CNVb.142" to "CNVb.173" in the manuscript.*


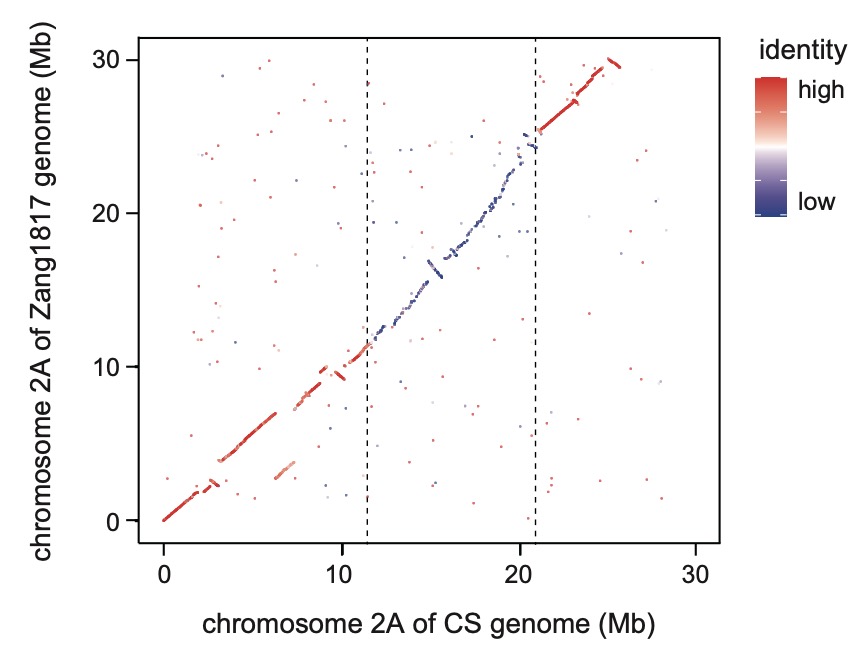


**Figure S10**. Comparative dot plot alignment between Zang1817 chromosome 2A and CS chromosome 2A (showing the first 30 Mb). The black dashed lines mark the regions where the CNVb.173 marker is located.

**Table R2.** BLAST search results for introgression segment on chromosome 2A of Zang1817. The table presents the top ten sequence matches from a Nucleotide BLAST search aimed at identifying potential species sources for the genomic segment found on chromosome 2A (11.5-21 Mb) of the Zang1817 wheat genome.

| **Scientific Name** | **Max Score** | **Total Score** | **Query Cover** | **E value** | **Per.Ident** |
| --- | --- | --- | --- | --- | --- |
| Triticum monococcum | 14704 | 1.7E+07 | 52% | 0.0 | 92.7 |
| Triticum monococcum | 15250 | 1.2E+07 | 50% | 0.0 | 97.4 |
| Triticum aestivum | 15027 | 6.2E+06 | 47% | 0.0 | 97.7 |
| Aegilops umbellulata | 13492 | 4.7E+06 | 45% | 0.0 | 90.5 |
| Triticum aestivum | 12597 | 4.4E+06 | 43% | 0.0 | 92.8 |
| Triticum aestivum | 13151 | 3.6E+06 | 48% | 0.0 | 96.6 |
| Triticum aestivum | 13740 | 2.6E+06 | 38% | 0.0 | 95.1 |
| Triticum aestivum | 12759 | 2.4E+06 | 39% | 0.0 | 95.8 |
| Triticum aestivum | 11215 | 2.2E+06 | 38% | 0.0 | 89.9 |
| Triticum urartu | 11880 | 2.1E+06 | 37% | 0.0 | 91.3 |

11. In the method for identifying CNV blocks, the authors utilized the criterion of read counts below 0.5 or above 1.5 to distinguish between deletion and duplication windows. It is crucial to clarify whether a specific assay was employed to establish this criterion. Furthermore, it is important to investigate whether there are specific criteria in place for determining copy numbers exceeding 2 and how these high-copy number blocks will be accessed in the marker identification process and genotyped in subsequent applications. Additionally, Line 531 prompts questions about the criteria used for genotyping CNVb markers using low-coverage data. It is essential to delve into the details of this genotyping criterion and evaluate its effectiveness under the constraints of low-coverage conditions.

***Response:***

*Thanks for your valuable suggestions. The related content has been therefore revised to provide more details. The criterion of read counts to distinguish between deletion and duplication windows based on the distribution pattern of normalized read counts. We derived our thresholds from a detailed analysis of normalized coverage values obtained from resequencing data of 528 accessions. By plotting density distribution histograms* *of these values, we observed a near-normal distribution centered around a value of 1* ***(Figure R9)****. This observation led us to establish thresholds below 0.5 for deletions and above 1.5 (including above 2) for duplications. This method is consistent with the approaches used to identify CNVs in previous studies [5]. We have revised and supplemented the Methods section to better explain the basis for choosing thresholds to distinguish between deletion and duplication windows as follows.*

*“According to the distribution pattern of normalized read counts, which showed a near-normal distribution centered around a value of 1, windows exhibiting normalized read counts below 0.5 or above 1.5 were classified as deletion and duplication windows, respectively.”*

*We classify blocks with copy numbers of 2 or higher as duplication blocks. To elucidate our approach for CNVb marker identification and genotyping, especially those corresponding to high-copy number blocks, we introduced a section titled “Identification of CNVb markers using ulcWGS” in the Methods as follows.*

*“The pipeline is to first identify the type of CNV blocks and then match these CNV blocks to the corresponding markers to identify which markers are present in each variety. Initially, the ulcWGS data are aligned to the pan-genome to detect raw deletion (copies=0) and duplication (copies≥2) blocks. These blocks are then mapped separately to their corresponding CNVb marker set—deletion blocks to the deletion marker set and duplication blocks to the duplication marker set. The presence of a deletion or duplication marker in a variety is determined based on the following criteria, if a deletion or duplication block present in the variety overlaps with a deletion or duplication marker by at least 90% and the difference in length between the block and the marker is less than 100 Kb, the deletion or duplication marker is considered present in that variety.”*

*This pipeline is visually represented in* ***Figure S4*** *for clarity.*

*We rigorously evaluated the effectiveness of our genotyping approach for low-coverage data by comparing the CNVb markers identified in 100 randomly selected varieties at* *0.05x sequencing coverage against markers identified at higher coverages in the manuscript. The comparison yielded an average F-score value exceeding 0.98 (****Fig. 4d****), affirming the reliability and accuracy of our low-coverage genotyping approach.*


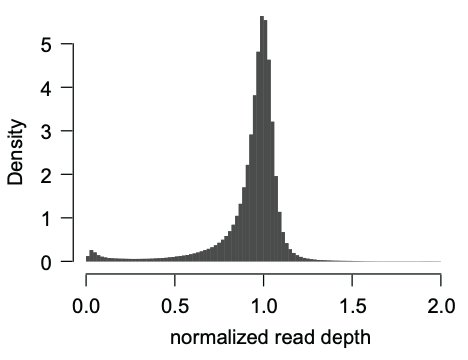


**Figure R9**. Histogram of normalized read depth for genomic bins across 528 accessions used in constructing CNVb markers. The histogram only shows the distribution for normalized read depths up to 2.

***Reference***

1. *Walkowiak S, Gao L, Monat C, et al. Multiple wheat genomes reveal global variation in modern breeding. Nature, 2020, 588(7837): 277–283.*
2. *Gao L, Jia J, Kong X. A SNP-Based Molecular Barcode for Characterization of Common Wheat. PLOS ONE, 2016, 11(3): e0150947.*
3. *Przewieslik-Allen A M, Wilkinson P A, Burridge A J, et al. The role of gene flow and chromosomal instability in shaping the bread wheat genome. Nature Plants, 2021, 7(2): 172–183.*
4. *Cheng H, Liu J, Wen J, et al. Frequent intra- and inter-species introgression shapes the landscape of genetic variation in bread wheat. Genome Biology, 2019, 20(1).*
5. *Yang Z, Wang Z, Wang W, Xie X, Chai L, Wang X, Feng X, Li J, Peng H, Su Z, et al. ggComp enables dissection of germplasm resources and construction of a multiscale germplasm network in wheat. Plant Physiol 2022, 188:1950-1965.*
6. *Golicz A A, Batley J, Edwards D. Towards plant pangenomics. Plant Biotechnology Journal, 2015, 14(4): 1099–1105.*
7. *Aury J-M, Engelen S, Istace B, et al. Long-read and chromosome-scale assembly of the hexaploid wheat genome achieves high resolution for research and breeding. GigaScience, 2022, 11.*
8. *Delorean E, Gao L, Lopez J F C, et al. High molecular weight glutenin gene diversity in Aegilops tauschii demonstrates unique origin of superior wheat quality. Communications Biology, 2021, 4(1).*
9. *Huang X, Zhu M, Zhuang L, Zhang S, Wang J, Chen X, Wang D, Chen J, Bao Y, Guo J, et al. Structural chromosome rearrangements and polymorphisms identified in Chinese wheat cultivars by high-resolution multiplex oligonucleotide FISH. Theor Appl Genet 2018, 131:1967-1986.*

**Second round of review**

**Reviewer 1**

All my comments have been answered. I didn't have further questions.

**Reviewer 2**

The revised manuscript has made significant strides in addressing previous concerns and enhancing the overall clarity of the content. However, to further improve the quality and readability of the manuscript, I would like to offer the following suggestions:
1. There are a few grammar errors present in the text that should be addressed. I recommend a thorough review of the entire manuscript to correct these errors. For example, in Line 278, the word "conferring" should be replaced with "conferred."
2. In Line 183, the statement "By profiling these CNVb markers across the genome, we showed that these CNVb markers are distributed across all chromosomes with a median of 59" lacks clarity. What is "median of 59" exactly mean?
3. In Line 149, is the "pan-reference genome" refers to "pan-genome reference" ?
4. The phrase "CNV blocks large segments" in Line 404 is ambiguous and requires clarification to improve comprehension.
5. Providing detailed explanations for the vertical and horizontal coordinates in Figure 4a is recommended to aid readers in understanding this figure.
6. It is advisable to label each subfigure in Figure 5 to facilitate easy reference to specific parts of the figure in the text, enhancing the overall accessibility of the visual data.

**Authors Response**

**Point-by-point responses to the reviewers’ comments:**

Reviewer #2:
The revised manuscript has made significant strides in addressing previous concerns and enhancing the overall clarity of the content. However, to further improve the quality and readability of the manuscript, I would like to offer the following suggestions:

*Response:
Thank you for your positive feedback on the revisions and your valuable suggestions for further improvement. We have carefully considered your recommendations and have made additional revisions to enhance the quality and readability of the manuscript.*

1. There are a few grammar errors present in the text that should be addressed. I recommend a thorough review of the entire manuscript to correct these errors. For example, in Line 278, the word “conferring” should be replaced with “conferred”.
*Response:
Thank you for your suggestion regarding grammar errors. We reviewed the entire manuscript and have corrected all the potential grammatical issues.
Especially, we have revised the sentence in Line 278 to use “conferred” instead of “conferring” as follows, “One is a CNVb-deletion marker, CNVb.189 (chr2A:0-24.7 Mb), which was detected in Jagger (Fig. 3f), and is linked to a 2NvS introgression from Aegilops ventricosa that conferred resistance to wheat blast and carried the rust disease resistance gene cluster (Lr37/Yr17/Sr38) [15].”*

2. In Line 183, the statement "By profiling these CNVb markers across the genome, we showed that these CNVb markers are distributed across all chromosomes with a median of 59" lacks clarity. What is "median of 59" exactly mean?
*Response:
Thank you for your comment. The term "median" was originally intended to indicate the mean and has now been corrected accordingly. To improve clarity, we have corrected the sentence in Line 183 as follows, “By profiling these CNVb markers across the genome, we observed that these CNVb markers are distributed across all chromosomes, with an average of 59 markers per chromosome (Additional file 2: Fig S5).”*

3. In Line 149, is the “pan-reference genome” refers to “pan-genome reference” ?
*Response:
Thank you for pointing out the confusion caused by our wording in line 149.
Yes. We have revised “pan-reference genome” to “pan-genome reference” in this sentence as* *follows, “To mitigate the potential bias of CNVb identification introduced by mapping against a single reference genome and to acquire a sufficient number of genetic markers, we constructed a pan-genome reference by iterative mapping of the whole-genome resequencing data against 16 de novo assembled reference genome sequences [15, 26, 29-33].”*

4. The phrase "CNV blocks large segments" in Line 404 is ambiguous and requires clarification to improve comprehension.
*Response:
Thank you for pointing out the ambiguity in Line 404. We have revised the sentence as follows, “In this study, we revealed that the high frequency and polymorphism of large CNV blocks in wheat make CNVb an effective DNA-based marker for efficient variety identification.”*

5. Providing detailed explanations for the vertical and horizontal coordinates in Figure 4a is recommended to aid readers in understanding this figure.

*Response:
Thank you for your suggestion. We have revised the figure legend of Figure 4a, so as to provide detailed explanations for the vertical and horizontal coordinates into aid readers in understanding this figure. The updated legend now runs as follows, “The CNVb marker fingerprint of Lunxuan 987. CNVb marker fingerprint consists of a QR-code-like two-dimensional matrix, with each cell representing a CNVb marker. All the markers are ordered by chromosomes, and are filled into the matrix by rows, from left to right, and from top to bottom. Two specific markers were highlighted by arrows with annotated descriptions as interspecific introgression or structural variation.”*

6. It is advisable to label each subfigure in Figure 5 to facilitate easy reference to specific parts of the figure in the text, enhancing the overall accessibility of the visual data.

*Response:
Thanks for your suggestion. We have labeled each subfigure in Figure 5 to facilitate easy reference to specific parts of the figure in the manuscript (Fig. 5), enhancing the overall accessibility of the visual data.*
